# Supplementary material for: Revealing the predictability of intrinsic structure in complex networks
Source: Nat Commun. 2020 Jan 29;11:574. doi: 10.1038/s41467-020-14418-6 (PMC6989503; doi:10.1038/s41467-020-14418-6)
Supplement: Supplementary file 1 — Supplementary Information [file 41467_2020_14418_MOESM1_ESM.pdf]

Supplementary Information for
"Revealing the Predictability of Intrinsic Structure in Networks"

J. Sun et al.

Supplementary Content

Supplementary Note 1: Data sets **2**

Supplementary Note 2: Compression algorithm **3**

Supplementary Note 3: Discussion on the linear relationship and the bin size **9**

Supplementary Note 4: The relationship between the leave-one-out method and other methods **12**

Supplementary Note 5: Link prediction algorithms used in the study **15**

Supplementary Note 6: Lack of linear correlation between the network's raw compression length and its structure predictability **17**

Supplementary Note 7: Quantify the performance of practical prediction algorithms relative to true optimal **18**

Supplementary Note 8: False or missing links identification through compression length **19**

Supplementary Note 9: Theory for network structure predictability **22**

Supplementary Note 10: Bounds of prediction precision **29**

Supplementary Note 11: Discussion on the data commercial value **47**

Supplementary References **49**

Supplementary Note 1: Data sets

In this study, we consider 12 representative real networks, all of which are undirected networks.

Biological networks:

- **C.elegans**: The neural network of Caenorhabditis elegans (C.elegans) [1] is downloaded from M.Newman Network collection (http://www-personal.umich.edu/mejn/netdata/). The original network is directed and weighted; here we treat it as a simple network by ignoring the directions and weights. The refined network contains 297 nodes with average degree 14.46.

- **Metabolic**: The metabolic network of C.elegans [2] is obtained from Link prediction group (http://linkprediction.org/index.php/link/resource/data). The original network is weighted with many self-loops. Again, we treat it as a simple network by ignoring the weights and removing self-loops. The refined metabolic network has 453 nodes with average degree 8.94.

- **PPI**: The protein-protein interaction network in budding yeast is obtained from Pajek datasets of Vladimir Batagelj and Andrej Mrvar [3]. It contains 2617 nodes with average degree 9.06.

- **E.coli**: The protein-protein interaction network in the Escherichia coli K-12 [4] is obtained from Kcore Lab (http://www-levich.engr.ccny.cuny.edu/webpage/hmakse/software-and-data/). This network has 761 n-odes with average degree 5.98.

- **Bio-CE-GT**: The genetic interaction network of C.elegans [5] obtained from Network Reposito-ry (http://networkrepository.com/index.php) contains 924 nodes with average degree 7.01.

Social networks:

- **Political blogs**: A network of the US political blogs [6] is obtained from M.Newman Network collection. The original links are directed, here we treat them as undirected links. There are in total 1224 nodes with average degree 27.3.

- **Facebook**: A network of small group of Facebook users [7] is downloaded from Stanford Large Network Dataset Collection (http://snap.stanford.edu/data/). It contains 786 users (nodes) with average number of friends (average degree) 35.68.

- **Soc-wiki-vote**: The Wikipedia voting network from the inception of Wikipedia till January 2008 [5] is downloaded from Network Repository. Nodes in the network represent wikipedia users. The original network is directed, here we treat it as undirected network with 889 nodes and average degree 6.56.

- **Econ-wm1**: An economic network [5] is obtained from Network Repository. Nodes in this network are different individual agents (firms, banks and countries) while links represent mutual interactions between nodes. The original network is weighted with self-loop. Here we ignore the weight of each of links and remove every self-loop, resulting in a simple undirected network with 277 nodes and average degree 17.19.

- **Econ-mahindas**: An economic network of Victoria, Australia in 1880 [5] is obtained from Network Repository. Again, we transform the original network with weighted links and self-loops to a simple undirected network with 1258 nodes and average degree 11.93.

Technical networks:

- **USAir**: The US Air transportation network [8] is obtained from Pajek datasets (http://vlado.fmf.uni-lj.si/pub/networks/data/default.htm). There are in total 332 airports (nodes) with average airline (average degree) 12.80.

- **Tech-routers**; An Internet network at the router level [5, 9] is obtained from Network Repository. The nodes of the network represent routers, and the links represent physical connections between them. It is an undirected network with 2113 nodes and average degree 6.27.

Supplementary Note 2: Compression algorithm

In this section, we give a detailed description of the compression algorithm used in this study, which encodes the network structure into two bit strings. Experimental results are presented on both artificial random networks and real networks. We also study the relationship between the compression length and the number of compressed nodes.

Description of compression algorithm

Now we describe the general framework of the compression algorithm. Our compression scheme is based on the Structural Zip (SZIP) algorithm proposed by Y.Choi in 2012 [10], which is a lossless compression of graph.

SZIP has been proven to obtain the shortest lossless compression length for random networks, and also provides outstanding performances in real networks [10]. Specifically, the network topological structure (i.e., the network's adjacency matrix) is first encoded into two different bit strings. Based on the two bit stings, a network can be reconstructed which is isomorphic to the original network, i.e., the network structure can be reconstructed while the node label can not. These two bit strings are further compressed by an improved arithmetic encoder [11].

For the sake of readability, here we give an example of network topological encoding as shown in Supplementary Figure 1: The nodes in the network is encoded one by one. We select a node arbitrarily, say a, and store the number of neighbors of a (i.e., the degree of a) in binary ('010' in row '1' of the forth column). Then we partition the remaining nodes into two sets according to whether they are neighbors of a ($\{b, c\}$ and $\{d, e, f, h, g\}$ in row '1' of the fifth column). So far the encoding of node a is finished. Then, we select a new node, say b, from the neighbors of a to encode. We store two numbers: the number of neighbors of b among each of the above two sets also in binary ('1' and '010' in row '2' of the forth column). Then the remaining nodes are further partitioned into four sets (see the row '2' of the forth column, an empty set is omitted) according to whether they are neighbors of a and b, and we finish the encoding of node b. After that another node from the first set ($\{c\}$) is chosen to be encoded in the similar way. This procedure continues until all nodes are processed. During the construction, the number of neighbors of the selected node with respect to each set in the partition is appended to either sequence B_1 or sequence B_2, where B_2 contains those numbers for singleton sets (i.e., we store either '0' when there is no neighbor or '1' otherwise). In the next subsection we will discuss the effect of node order on the ultimate compression length.

In the second stage, we use an arithmetic encoder to compress $\mathbf{B}_1, \mathbf{B}_2$ separately. Particularly, we employ an improved arithmetic encoder based on recursive splitting proposed by K.Skretting [11]. By exploiting the dependencies between the symbols of the bit sting, such encoder can further enhance the compression performance compared to straightforward arithmetic encoder [12]. Finally we obtain two compressed binary sequences $\hat{\mathbf{B}}_1$, $\hat{\mathbf{B}}_2$, and we define L to be the sum of the length of these two sequences:

$$L = \ell(\hat{\mathbf{B}}_1) + \ell(\hat{\mathbf{B}}_2), \tag{1}$$

where $\ell(\hat{\mathbf{B}}_1)$,$\ell(\hat{\mathbf{B}}_2)$ are the length of $\mathbf{B}_1$ and $\mathbf{B}_2$, respectively.

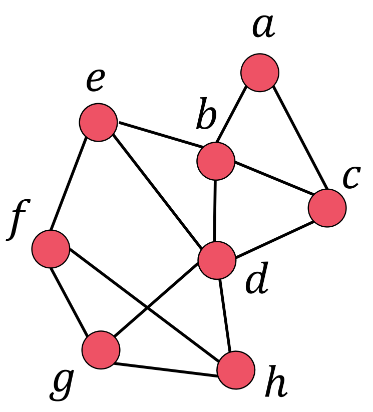

t	v	$P_{t-1} - v$	encoding	P_t
0				$abcdefgh$
1	a	$bcdefgh$	010	$bc/defgh$
2	b	$c/defgh$	1, 010	$c/de/fgh$
3	c	de/fgh	01, 00	$d/e/fgh$
4	d	e/fgh	1, 10	$e/gh/f$
5	e	gh/f	00, 1	gh/f
6	g	h/f	1, 1	h/f
7	h	f	1	f
8	f			

Supplementary Figure 1. An example of the topological encoding. Let v be the chosen node to be encoded in the t step and $\mathbf{P}_t$ be the partition of the set of remaining nodes after the t step. For each of subsets $\mathbf{U}$ in each line of the third column $\mathbf{P}_{t-1} - v$, we use $\lceil \log(|\mathbf{U}| + 1) \rceil$ bits to encode the number of neighbors of v in $\mathbf{U}$, where $\lceil x \rceil$ means that x is rounded up to the nearest integer. During the encoding process, those of length more than one bit (i.e., $|\mathbf{U}| > 1$) are appended to sequence $\mathbf{B}_1$, while those of length exactly one bit (i.e., $|\mathbf{U}| = 1$) are appended to sequence $\mathbf{B}_2$. After 8 steps, $\mathbf{B}_1$ and $\mathbf{B}_2$ are 01001001001000 and 11111, respectively.

Robustness of compression results

To test our algorithm, we apply it to both artificial random networks and real-world networks. Notably, there can be different choices at each step for selecting the next node during the topological encoding process described in Supplementary Note . Here, we show that node ordering during encoding does not affect the ultimate compression length L, as shown in Supplementary Figure 2. Here we consider three different kinds of order: (i) the ascending order by the node's degree (That is, we first choose the node a with the maximal degree in the network to encode. Then a node having the maximal degree among the neighbors of a is chosen to be encoded). (ii) the descending order by the node's degree and (iii) the purely random order. We find that in all of the studied real networks including their shuffled versions (the shuffling operation randomly picks f fraction of links, and rewires them to randomly selected pairs of nodes), there is no substantial difference among the three ordering methods. In other words, the compression length L remains robust for the selection of node order. Without loss of generality, we adopt random order in this paper and present the compression results on their average and the standard deviation.

We now summarise the compression performance for the real networks. For comparison, we list the lengths of four other encoding of networks, namely, (i) the simple representation of the adjacency matrix. Since the network

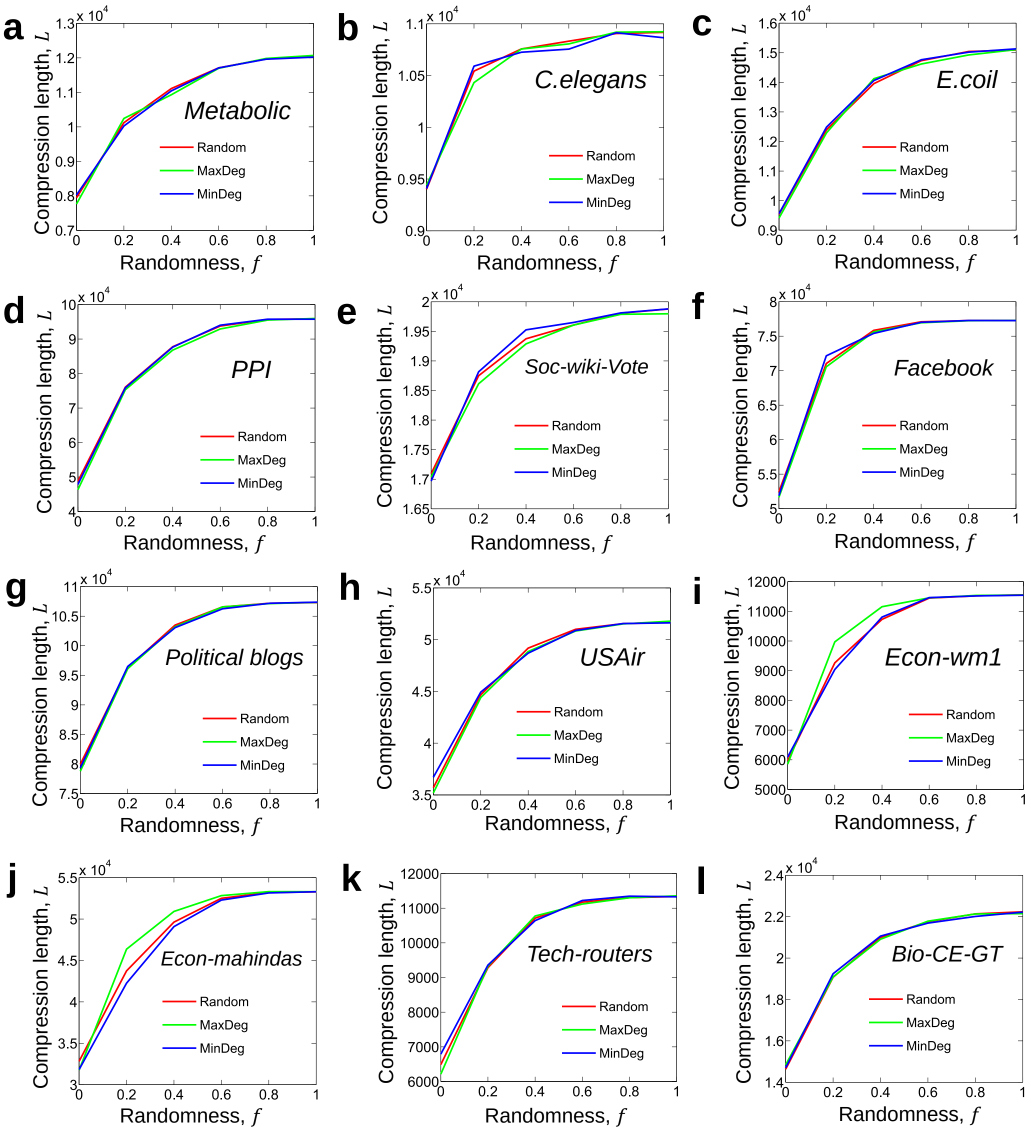

Supplementary Figure 2. Compression length L vs degree of randomness f for different node selection orders during the topological encoding. Note that $f = 0$ refers to the original real-world network, while $f = 1$ refers to the entirely random (ER) network with the same number of nodes and links. "MaxDeg" and "MinDeg" denote that nodes are selected in increasing and decreasing order of node degree during the topological encoding, respectively. While nodes are selected with random in the "Random" method, whose result is the average value of 50 independent experiments.

Supplementary Table 1. The average bitstring length for real-world networks. Nodes are selected randomly during the topological encoding stage. All the numbers are averages over 50 measurements. The entries corresponding to the minimal compression length among these 5 methods are emphasized in black.

Network	Average Compression Length (bits)				
	Adj. mat $\binom{N}{2}$	Arithmetic	Recur. arithmetic	Topological+ arithmetic	Topological+ recur. arithmetic
USAir	54946	12998	11046	8137	**6484**
C.elegans	43956	12394	10653	9682	**9401**
Metabolic	102378	14372	12953	8286	**7944**
PPI	3423036	113995	99591	58561	**48781**
E.coli	289180	19198	18186	10516	**9541**
Political blogs	748476	115541	90503	96620	**79721**
Facebook	308505	82323	78877	62840	**52412**
Bio-CE-GT	426426	27480	19739	15889	**14630**
Soc-wiki-vote	394716	24845	21811	17655	**17094**
Econ-wm1	38226	12936	6660	7486	**5697**
Econ-mahindas	790653	61295	42616	38023	**32824**
Tech-routers	2231328	65247	65267	40152	**35697**

is represented by a undirected graph, we only need the upper/lower triangle of the adjacency matrix, resulting in $\binom{N}{2}$ bits. We then apply (ii) a standard arithmetic encoder [12] and (iii) the recursive arithmetic encoder [11] to the adjacency matrix respectively. Finally, we consider (iv) the original SZIP algorithm[10], i.e., encoding the network into two binary sequences and then compressing them using a standard arithmetic encoder. As illustrated by Supplementary Table 1, for each of real-world networks, our compression algorithm achieves shorter length than other methods.

The compression length of ER networks plays the role of normalization constant of the compression length of real-world networks with the same network size and average degree. Now we present the compression performance on ER random networks. In [10], Choi *et al* have proven that, for the ER networks with the size N and the probability q having a link between any pair of nodes, the theoretical compression length $\mathcal{R}$ obtained by SZIP can asymptotically achieves the structural entropy:

$$\mathcal{R} = \binom{N}{2} h(q) - N \log N \tag{2}$$

where $h(q) = -q\log q - (1-q)\log(1-q)$ is the binary entropy. Note that $\log$ represents the logarithmic operation of base $= 2$ throughout this paper.

To compare the experimental and theoretical results, Supplementary Figure 3 shows the difference between the

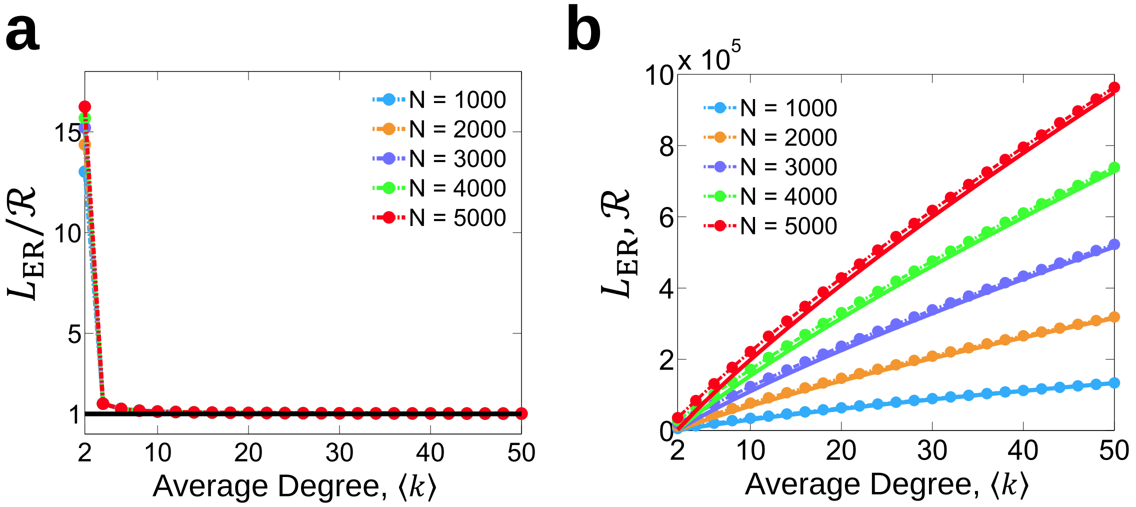

Supplementary Figure 3. Compression performance for ER networks. **a** Dependence of $L_{\rm ER}/\mathcal{R}$ on average degree $\langle k \rangle$ with varying network size N. **b** The relation between compression length and $\langle k \rangle$ with varying network size N. The circles with dashed line represent experimental result $L_{\rm ER}$ and the solid lines represent theoretical result $\mathcal{R}$. Each data point is the average result over 50 network instances.

experimental length $L_{\rm ER}$ and the theoretical length $\mathcal{R}$ on many ER networks with varying network size N and average degree $\langle k \rangle$. We can see that the real compression length indeed can achieve the theoretical value when $\langle k \rangle$ is large. However, the disagreement between $L_{\rm ER}$ and $\mathcal{R}$ appears when $\langle k \rangle$ is small (roughly less than 5). Therefore, to accurately estimate the structural entropy of a network with average degree less than 5, we use the experimental compression length $L_{\rm ER}$ instead of $\mathcal{R}$ to calculate the normalized compression length L^*, i.e.:

$$L^* = \frac{L}{L_{\rm ER}} \tag{3}$$

For networks with average degree equal to or larger than 5, we calculate L^* by the theoretical value $\mathcal{R}$ since $\mathcal{R}$ is nearly equivalent to $L_{\rm ER}$ in these networks. For better readability, we only present $L^* = \frac{L}{\mathcal{R}}$ as Eqn. (1) in the main text.

Impact of number of compressed nodes on the compression length

In this subsection we present the relationship between the compression length and the number of encoded nodes. We define $\mathbf{B}_1^t, \mathbf{B}_2^t$ to be bit strings $\mathbf{B}_1$, $\mathbf{B}_2$ when encoding the subnetwork consisting of randomly selected t nodes. Again, $\mathbf{B}_2^t$ contains those numbers for singleton sets (i.e., '0' or '1'). We then employ the recursive arithmetic encoder to compress $\mathbf{B}_1^t, \mathbf{B}_2^t$ respectively, resulting in compressed bit stings $\hat{\mathbf{B}}_1^t, \hat{\mathbf{B}}_2^t$. Similar to the definition of

L, we denote the sum of lengths of $\hat{\mathbf{B}}_1^t, \hat{\mathbf{B}}_2^t$ to be L^t. In Supplementary Figure 4, we show the relation between L^t and the size of the subnetwork with t nodes. One can see that when t is relatively large, the fluctuation of the network compression length is small.

Supplementary Note 3: Discussion on the linear relationship and the bin size

In this section, we discuss the effect of bin size on the linear relation between the normalized compression length L^* and the normalized BPAA performance entropy H^*_{BPAA}. As described in the main text, to measure the ranking distribution of link $\mathbf{D}$, where each r_i can vary in the range $1 \le r_i \le \frac{N(N-1)}{2} - \frac{\langle k \rangle N}{2} + 1 \approx \frac{N^2}{2}$, we divide such range into bins with equal width N to avoid the contribution of network size on the result. This leads to the probability distribution $\{p_j\}$ of these $\frac{N^2/2}{N} = N/2$ bins, where p_j is the probability that the missing link is in bin j. By calculating the BPAA performance entropy $H_{\mathrm{BPAA}} = -\sum_{j=1}^{N/2} p_j \mathrm{log} p_j$ and further normalizing it to H^*_{BPAA}, we can obtain the fitting linear relation between H^*_{BPAA} and L^*:

$$H^*_{\mathrm{BPAA}} \approx 1.63 L^* - 0.63 \tag{4}$$

In fact, one can extend Supplementary Equation 4 to the case with higher resolution of the ranking distribution that each bin is further divided into ℓ equispaced sub-bins. Approximately, we assume that when dividing the jth bin into ℓ sub bins, each sub bin still have the probability of missing link p_j, same as the jth bin. For example, we partition the probability p_1 of the first bin with range $[1, N)$ into ℓ bins each with equal probability of $\frac{p_1}{\ell}$. Recall that the entropy of the original probability distribution $\{p_j\}$ with bin length N is given by:

$$H = -\sum_{j=1}^{N/2} p_j \mathrm{log} p_j \tag{5}$$

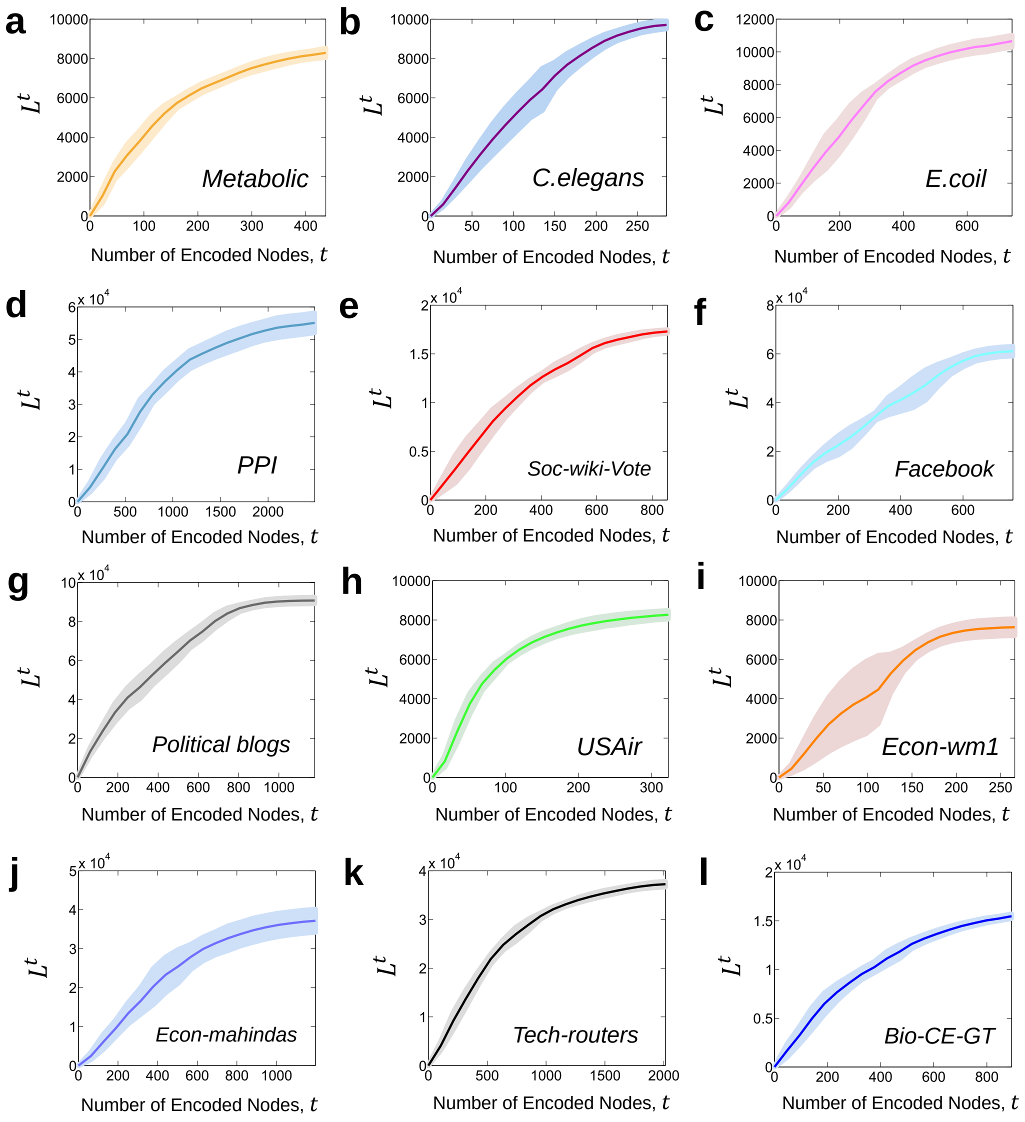

Supplementary Figure 4. The relation between the compression length and the number of encoded node. Within each of real-world networks, nodes are selected with pure random during the topological encoding. Each line is an average of 50 simulations and the shaded region is the bounded by the standard deviation.

For better readability, here we denote $\grave{H}$ as the high resolution entropy function of probability with bin length N/ℓ, then the relation between the $\grave{H}$ and H is given by:

$$
\begin{aligned}
\grave{H} &= -(\ell\frac{p_1}{\ell}\log\frac{p_1}{\ell} + \ell\frac{p_2}{\ell}\log\frac{p_2}{t} + ... + \ell\frac{p_{N/2}}{\ell}\log\frac{p_{N/2}}{\ell}) \\
&= -\sum_{j=1}^{N/2} p_j\log\frac{p_j}{\ell} \\
&= -\sum_{j=1}^{N/2}(p_j\log p_j - p_j\log\ell) \\
&= -\sum_{j=1}^{N/2} p_j\log p_j + \log\ell \\
&= H + \log\ell
\end{aligned} \tag{6}
$$

Recall that in the main text we define the following normalized BPAA performance entropy H^*_{BPAA} as:

$$H^*_{\mathrm{BPAA}} = \frac{H_{\mathrm{BPAA}}}{\log\frac{N}{2}} \tag{7}$$

Again, H_{BPAA} denotes the minimal value of H obtained by the BPAA. Similarly, here we define the normalized BPAA high resolution entropy $\grave{H}^*_{\mathrm{BPAA}}$ as:

$$\grave{H}^*_{\mathrm{BPAA}} = \frac{\grave{H}_{\mathrm{BPAA}}}{\log\frac{N\ell}{2}} \tag{8}$$

where the numerator represents the minimal $\grave{H}$ obtained by the BPAA, and the denominator represents the entropy of ER network where the ranking range of r_i is divided into $\frac{N\ell}{2}$ bins. By combing Supplementary Equations 4-8, we have:

$$\grave{H}^*_{\mathrm{BPAA}} \approx \frac{\log\frac{N}{2}}{\log\frac{N}{2} + \log\ell} * 1.63L^* - \frac{\log\frac{N}{2}}{\log\frac{N}{2} + \log\ell} * 0.63 + \frac{\log\ell}{\log N\ell - 1} \tag{9}$$

Notably the slope and the intercept are dependent on the network size N. We can see that, however, $\log N \gg \log\ell$ when N tends to infinity. Supplementary Figure 5a-b illustrate the value of $\grave{H}^*_{\mathrm{BPAA}}$ vs L^* in Supplementary Equation 9 for $\ell = 2, \ell = 4$ respectively. We can observe that the effect of the specific parameter N on the slope and the intercept of the line is not substantially significant, and Supplementary Equation 9 can reliably reflect the linear relation between the normalized algorithm performance entropy $\grave{H}^*_{\mathrm{BPAA}}$ and normalized shortest compression length L^*.

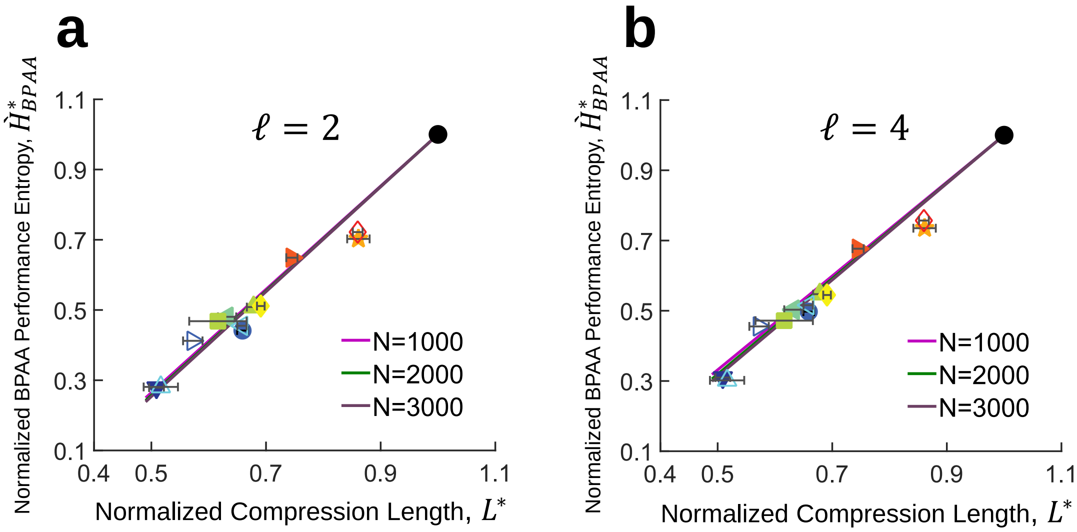

Supplementary Figure 5. Value of $\grave{H}^*_{\mathrm{BPAA}}$ vs L^* for **a** $\ell = 2$ and **b** $\ell = 4$. Each of dots represents a real-world network as the same in the main text.

Supplementary Note 4: The relationship between the leave-one-out method and other methods

In this section, we discuss the relationship between the leave-one-out method [13, 14] used in this paper and the other existing methods [15], which remove x fraction of links (x is usually 5% or 10%), and use the remaining links to calculate a score for the links not present in the remaining network. In the leave-one-out method, we only remove 1 link at a time, and calculate its ranking value. After calculating the ranking of each link in this way, the distribution entropy of the ranking is used as the structure predictability by us. Specifically, for a network with 1000 links, we need to calculate the ranking 1000 times. For existing methods, every time x fraction of links is removed, the ranking only needs to be done once for the removed links together. Hence if x is chosen to be very close to 0, and distribution entropy is measured by repeatedly sampling, it becomes similar to the leave-one-out method as well. Effectively, this is approximately a sub sample in our 1000 sample points. Qualitatively, doing so would yield similar results as our method by removing 1 link at a time as shown in Supplementary Figure 6 below. It shows that on the Metabolic and USAir networks using BPAA algorithm, our method has the similar ranking distribution and its entropy value with the repeated sampling for removing x fraction of links. As the fraction of removed links decreases from 10% to 1%, the result is approaching ours. However, without repeated sampling on removing x fraction, the fluctuation in distribution is much larger as shown in Supplementary Figure

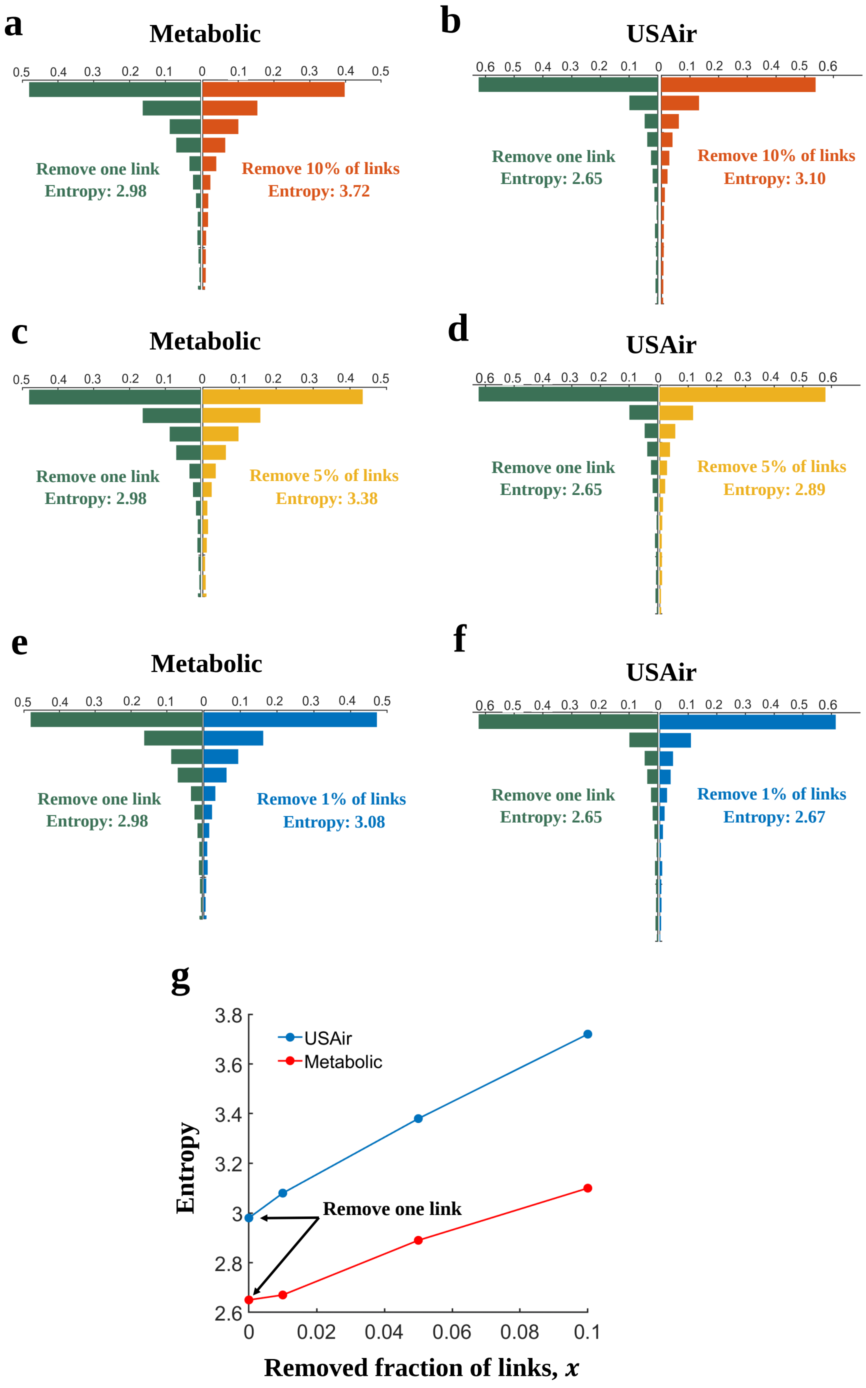

Supplementary Figure 6. Leave-one-out method and the other popular methods in measuring algorithm performance entropy. For other methods, we used multiple samples of removing x fraction of links to obtain the average behaviors. As the fraction of removal decreases (10%-5%-1%), the entropy value decreases and approaches the leave-one-out method for removing 1 link at a time.

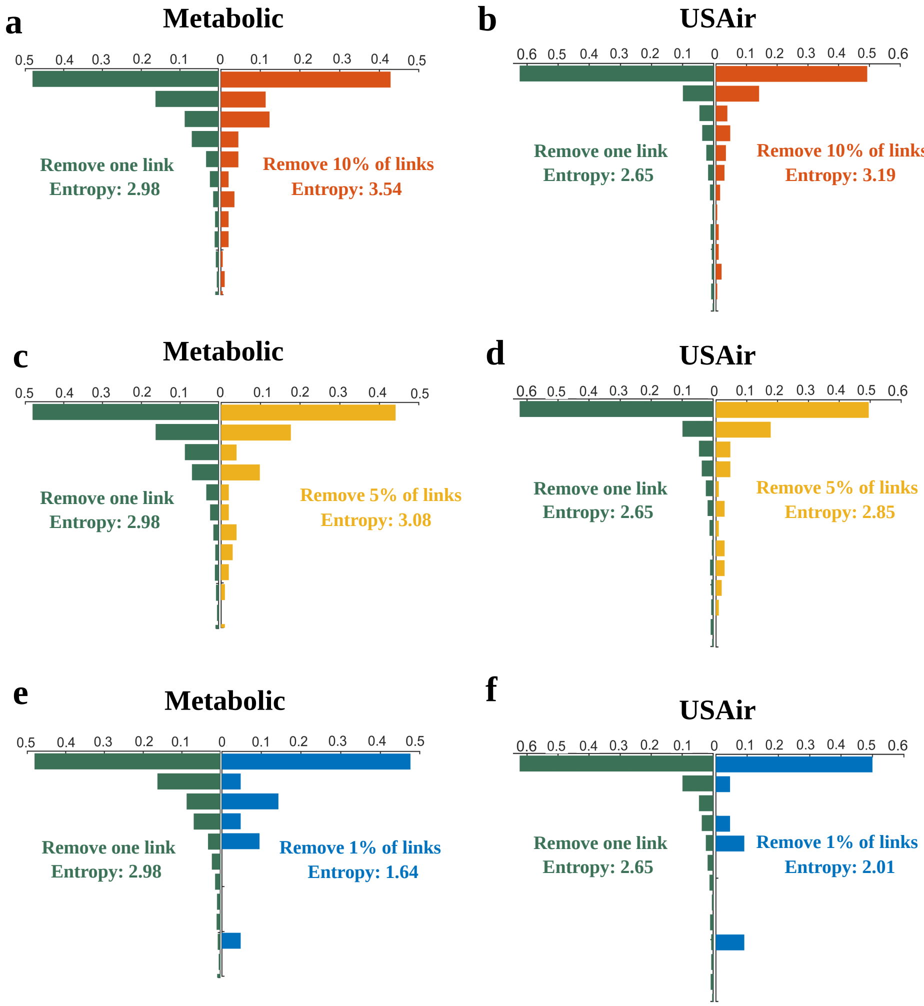

Supplementary Figure 7. Similar to the Supplementary Figure 6, but here we only use 1 sample of removing x fraction. The fluctuation in ranking values is significant, and more pronounced for small x.

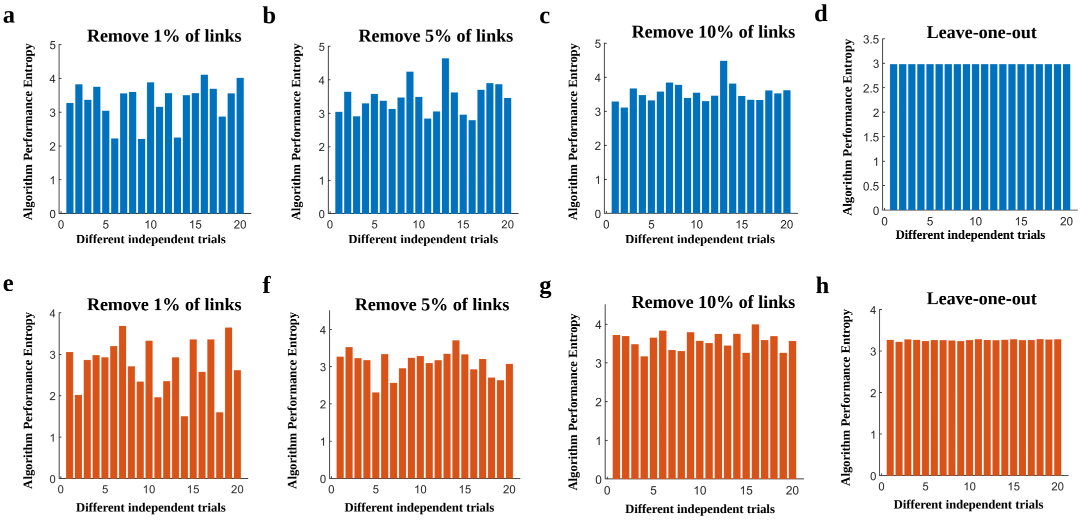

Supplementary Figure 8. Algorithm performance entropy in Metabolic network obtained by different methods. **a-d** RA algorithm, which is a deterministic algorithm. **e-h** SPM algorithm, which involves randomness.

7. Even though it is possible to remove more than 1 link at a time, removing different batches of links would result in different performance entropy values as shown in Supplementary Figure 8a-c and e-g. Such fluctuation would result in uncertainty in the results without clear advantage. On the other hand, leave-one-out method has minimal fluctuation in the results as shown in Supplementary Figure 8d and h, while preserving the original network as much as possible. This facilitates us to find more accurate linear relationship between the network compression length and predictability.

Supplementary Note 5: Link prediction algorithms used in the study

The key of link prediction problem is to assign a score for each unobserved link using observed network structure. The higher the score, the larger is the likelihood of existence of unobserved links [16, 15]. There are many different types of algorithms aiming at solving the link prediction problem. Here we give a brief description of the 11 popular algorithms used in this study. For convenience, for a node x, let $\mathbf{\Gamma}(x)$ and $k(x)$ denote the set of neighbors of x and the degree of node x, respectively.

- Structural Perturbation Method (SPM) [17]

  The SPM method assumes that the regularity of a network is reflected in the consistency of structural features before and after a random removal of a small set of links. Based on the perturbation of the adjacency matrix,

a structural consistency index is defined as:

$$s_{xy}^{\mathrm{SPM}} = \tilde{\mathbf{A}}_{xy} \tag{10}$$

where $\tilde{\mathbf{A}}$ represents the perturbed matrix constructed via first-order approximation. More detailed derivation can be found in [17].

- Local Random Walk (LRW) [18]

  To measure the similarity between nodes x and y, a random walker is initially put on node x and thus the initial density vector $\vec{\pi}_x(0) = \vec{e}_x$. This density vector evolves as $\vec{\pi}_x(t+1) = \mathbf{P}^T \vec{\pi}_x(t)$ for $t \geq 0$. The LRW index at time step t is thus defined as:

$$s_{xy}^{\mathrm{LRW}}(t) = q_x \pi_{xy}(t) + q_y \pi_{yx}(t). \tag{11}$$

  where q is the initial configuration function.

- Superposed Random Walk (SRW) [18]

  Similar to the LWR index, Liu and Lu [18] proposed the SRW index, where the random walker is continuously released at the starting point, resulting in a higher similarity between the target node and the nodes nearby. The mathematical expression reads

$$s_{xy}^{\mathrm{SRW}}(t) = \sum_{\tau=1}^{t} s_{xy}^{\mathrm{LRW}}(\tau) = \sum_{\tau=1}^{t} [q_x \pi_{xy}(\tau) + q_y \pi_{yx}(\tau)]. \tag{12}$$

- Average Commute Time (ACT) [19]

  Assuming two nodes are more similar if they have a smaller average commute time, which can be measured by the average number of steps required by a random walker, thus the ACT index can be defined as

$$s_{xy}^{\mathrm{ACT}} = \frac{1}{l_{xx}^{+} + l_{yy}^{+} - 2l_{yy}^{+}} \tag{13}$$

  where l_{xy}^{+} denotes the corresponding entry in the pseudoinverse of the Laplacian matrix $\mathbf{L}^{+}$ [19, 20].

- Common neighbors (CN) [16]

  In common sense, two nodes, x and y, are more likely to have a link if they have many common neighbors. Therefore, the simplest measure of this neighborhood overlap is the directed count, namely

$$s_{xy}^{\mathrm{CN}} = |\mathbf{\Gamma}(x) \bigcap \mathbf{\Gamma}(y)| \tag{14}$$

- Length of 3 (L3) index [21]

  It is defined as:

$$s_{xy}^{\mathrm{L3}} = \sum_{u,v} \frac{A_{xu} A_{uv} A_{vy}}{\sqrt{k_u k_v}} \tag{15}$$

  where $A_{xu} = 1$ if nodes x and u are connected and $A_{xu} = 0$ otherwise. k_u is the degree of node u.

- Jaccard index [22]

  It is defined as:

$$s_{xy}^{\mathrm{Jaccard}} = \frac{|\mathbf{\Gamma}(x) \bigcap \mathbf{\Gamma}(y)|}{|\mathbf{\Gamma}(x) \bigcup \mathbf{\Gamma}(y)|} \tag{16}$$

- Salton index [23]

  It is defined as:

$$s_{xy}^{\mathrm{Salton}} = \frac{|\mathbf{\Gamma}(x) \bigcap \mathbf{\Gamma}(y)|}{\sqrt{k(x) \times k(y)}} \tag{17}$$

- Preference Attachment (PA) index [24, 25]

  It is defined as:

$$s_{xy}^{\mathrm{PA}} = k(x) \times k(y) \tag{18}$$

- Adamic-Adar allocation (AA) [26]

  It is defined as:

$$s_{xy}^{\mathrm{AA}} = \sum_{z \in \mathbf{\Gamma}(x) \bigcap \mathbf{\Gamma}(y)} \frac{1}{\log |\mathbf{\Gamma}(z)|} \tag{19}$$

- Resource allocation (RA) [27]

  It is defined as:

$$s_{xy}^{\mathrm{RA}} = \sum_{z \in \mathbf{\Gamma}(x) \bigcap \mathbf{\Gamma}(y)} \frac{1}{|\mathbf{\Gamma}(z)|} \tag{20}$$

Supplementary Note 6: Lack of linear correlation between the network's raw compression length and its structure predictability

In this section, we investigate the possibility of directly using network compression length without proper normalization to gauge predictability of networks. As illustrated in Supplementary Figure 9a, we plot the predictability (normalized algorithm performance entropy) from the best of the 11 algorithms vs. network compression length.

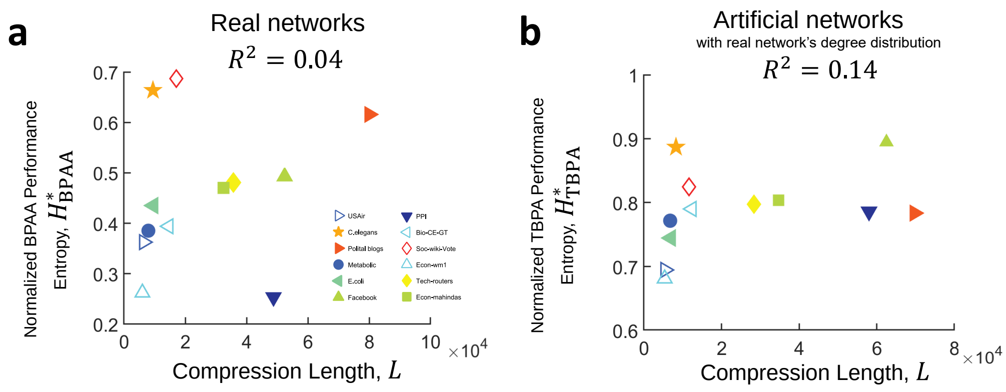

Supplementary Figure 9. Network direct compression length and network predictability measured by the normalized entropy. The result on 12 real and artificial networks show lack of significant correlation between the two quantities.

We find that, in real networks, there is almost no correlation between these two quantities ($R^2 = 0.04$). Additionally, we carried out the same analysis on artificial networks, which have the same degree distributions as the real ones, finding again a modest correlation at best (Supplementary Figure 9b, $R^2 = 0.14$). These results are in sharp contrast to the clear linear relationship we uncovered in our paper. Similarly, we also failed to find any meaningful correlation between the raw compression length and the unnormalized algorithm performance entropy (Supplementary Figure 10). These results demonstrate that, without the theoretical intuition on proper normalization, the network compression length itself does not serve as a good indicator of predictability.

Supplementary Note 7: Quantify the performance of practical prediction algorithms relative to true optimal

Based on the linear relationship between the normalized compression length and predictability, we can, in principle, quantify the performance of any prediction algorithm. Supplementary Figure 11 compares the Jaccard algorithm [35] performance entropy with the linear relationship. It can be seen that for any network, the further is the Jaccard algorithm performance entropy from the linear line, the less optimal it is from the best possible prediction results. This quantifiable distance can serve as a benchmark for any practical algorithm in any networks. In other words, the further it is from the linear relationship, the higher the potential of improvement for a new algorithm

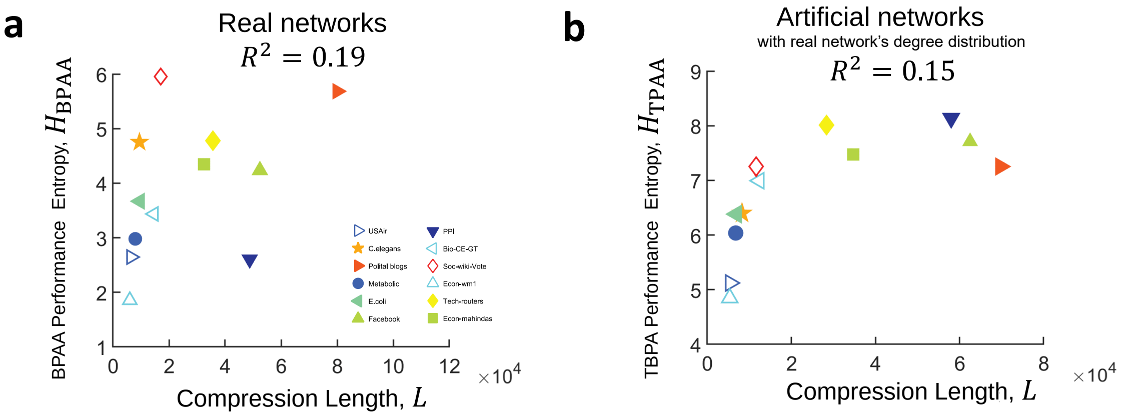

Supplementary Figure 10. Network direct compression length and network predictability measured the direct entropy of ranking distribution. The result on real and artificial networks still show lack of significant correlation between the two quantities.

to achieve. Conversely, if the algorithms performance entropy lies close to the line, it means that the algorithm is already performing quite well, and further exploration on algorithms is unlikely to yield significant improvement, suggesting diminishing returns in developing new algorithms.

Supplementary Note 8: False or missing links identification through compression length

An additional potential benefit of our finding is to improve algorithms in identifying missing or false links. If there is a small fraction of false links or missing links, it is possible to identify such links using network structural entropy (the shortest compression length). It should be noted that the purpose of link prediction algorithm is to identify the missing links or false links. For instance, the ranking of an existing link according to an algorithm can be related to how likely this link is a false link: the lower is the rank, the more likely it is false. To develop an algorithm using our result, we carry out the following initial study. We randomly add F links (e.g. $F = 100$) as false links to an original network, and also choose another T existing links (e.g. $T = 900$). From the seed set of $F + T = 1000$ links, we try to solely use the network compression length (structural entropy) as an indicator to identify these 100 false links. We use the following simple method: we remove each one of the 1000 links individually, and calculate the compression length of the modified network. The 100 removed links that lead to

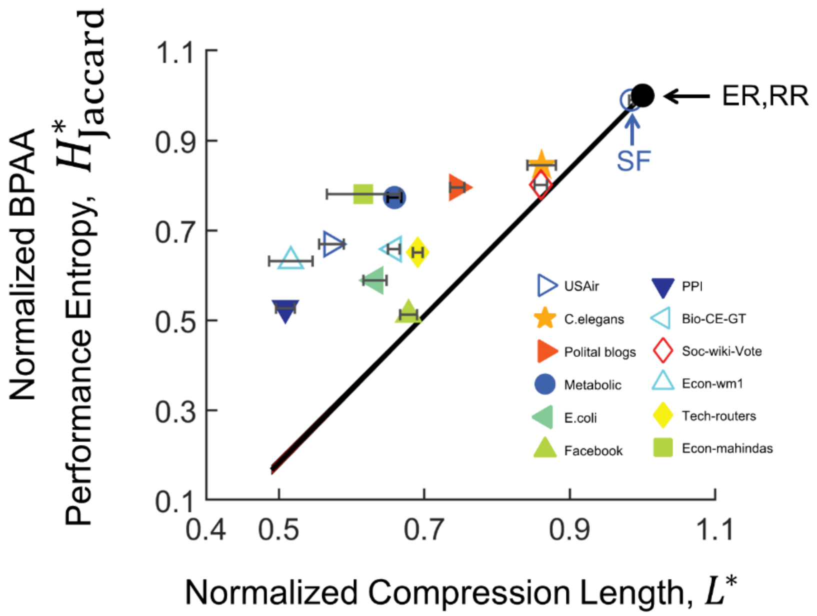

Supplementary Figure 11. Normalized performance entropy using Jaccard algorithm vs. compression length over different real-world networks.

the shortest compression length L are chosen to be the most likely false links, as random links lead to the longest compression lengths. In Supplementary Figure 12a-b, we see that this simple method is clearly outperforming random guess. Its accuracy is not very high at the moment, but we believe with more refinement and combination with more complicated techniques like simulated annealing, genetic algorithm etc., the accuracy can be further improved.

Similarly, we use the same method to identify missing links. In a network with full information on the existing links, we randomly remove $F = 100$ links as missing links. At the same time, we chose $T = 900$ links that are not present. Every time we add one link from the 1000 links, and calculate the network's compression length. The 100 links that give the shortest compression lengths are deemed as the missing links. From Supplementary Figure 12c-d, we see that this simple method still outperforms random guess. The above results imply that, network compression length (structural entropy) can be used as an independent indicator to identify missing or false links.

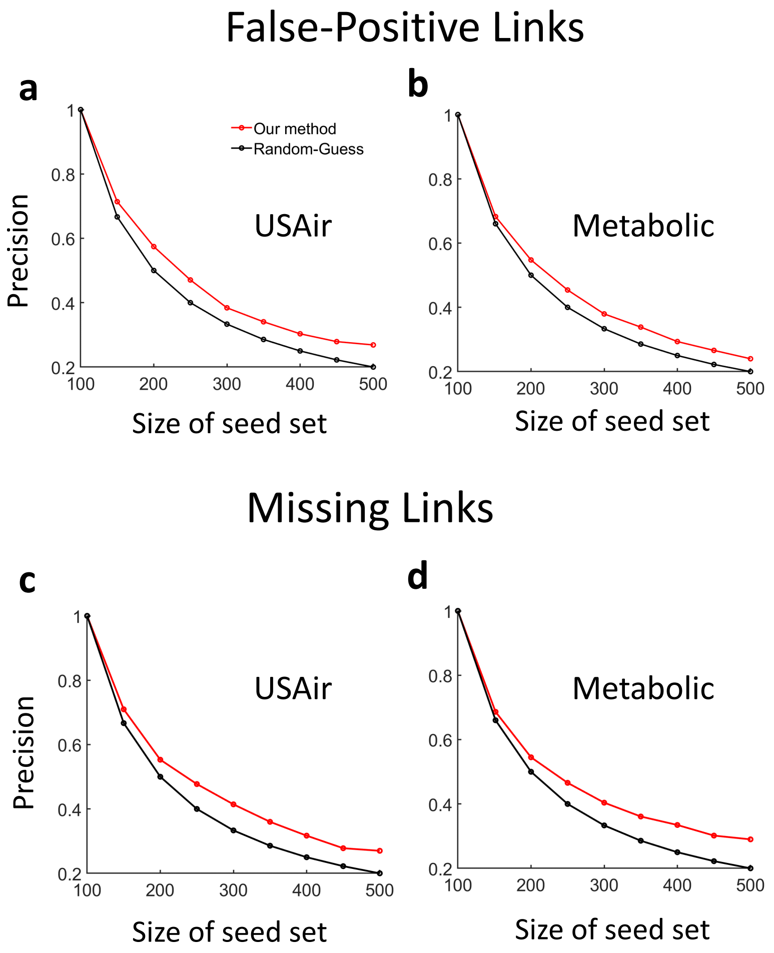

Supplementary Figure 12. Using structural entropy as indicator to identify **a-b** false links and **c-d** missing links.

Supplementary Note 9: Theory for network structure predictability

In this section, we provide a detailed derivation of the theoretical linear relationship between the shortest compression length and the link prediction limit. As stated in the main text, we assume that an artificial network is generated from a static random matrix $\mathbf{Q}$ whose entry q_{ij} denotes the link formation probability between node i and j.

Shortest compression length

According to Shannon's source coding theorem, the shortest compression length L of this artificial network should be equivalent to its structural entropy, yielding:

$$\begin{aligned} L &= \sum_{i>j} h(q_{ij}) - N\text{log}N \\ &= -\sum_{i>j} q_{ij}\text{log}q_{ij} - \sum_{i>j}(1-q_{ij})\text{log}(1-q_{ij}) - N\text{log}N \end{aligned} \tag{21}$$

where N is the network size. Note that Supplementary Equation 21 holds based on the following lemma:

Lemma 1 [28]: For all q satisfying $\frac{\text{ln}N}{N} \ll q$ and $1-q \gg \frac{\text{ln}N}{N}$, a random graph is symmetric with probability $O(N^{-w})$ for any positive constant w.

By substituting q with $\frac{\langle k \rangle}{N}$, we arrive at the estimated range: $\text{ln}N \leq \langle k \rangle \leq N - \text{ln}N$, where the theory is valid. Since $\langle k \rangle \leq N - \text{ln}N$ generally holds given that real networks are sparse networks, we only illustrate the estimated bound $\langle k \rangle = \text{ln}N$ denoted by a red curve in the Fig. 3 e in the main text.

By replacing the base of $\text{log}(1-q_{ij})$ to the mathematical constant e in Supplementary Equation 21, one can expand it as a Taylor series and neglect higher order terms given that q_{ij} is small, i.e.,

$$\text{log}(1-q_{ij}) = \frac{\text{ln}(1-q_{ij})}{\text{ln}2} = \frac{-q_{ij} - \frac{q_{ij}^2}{2} + \frac{-q_{ij}^3}{3} + ...}{\text{ln}2} \approx \frac{-q_{ij}}{\text{ln}2} \tag{22}$$

where ln represents the natural logarithmic of base $= e$. By Supplementary Equation 22, L can be approximated as

$$\begin{aligned} L &\approx -\sum_{i>j} q_{ij}\text{log}q_{ij} + \frac{1}{\text{ln}2}\sum_{i>j}(1-q_{ij})q_{ij} - N\text{log}N \\ &\approx -\sum_{i>j} q_{ij}\text{log}q_{ij} + \frac{1}{\text{ln}2}\sum_{i>j}(q_{ij} - q_{ij}^2) - N\text{log}N \\ &\approx -\sum_{i>j} q_{ij}\text{log}q_{ij} + \frac{1}{\text{ln}2}\sum_{i>j} q_{ij} - N\text{log}N \end{aligned} \tag{23}$$

For an undirected network generated from $\mathbf{Q}$ with number of nodes N and the average degree $\langle k \rangle$, it is clear that the sum of q_{ij} within the upper triangle of $\mathbf{Q}$ should be approximately equal to the total number of links in the generated network, i.e.,

$$\sum_{i>j} q_{ij} = \frac{N\langle k \rangle}{2} \tag{24}$$

By combining Supplementary Equations23,24, we could write

$$L \approx -\sum_{i>j} q_{ij} \log q_{ij} - N \log N + \frac{N\langle k \rangle}{2 \ln 2} \tag{25}$$

We then normalize L by the theoretical maximum compression length $\mathcal{R}$ through the same way of Eqn. 1 in the main text, yielding:

$$L^* = \frac{L}{\mathcal{R}} \tag{26}$$

Structural symmetry

According to Ref.[28], the probability for a network to have symmetrical structure $\mathbf{G}$ is upper bounded by $O(N^{-w})$, for arbitrarily small positive value w. Thus the possibility will approach to 0 when N is large enough, and it is no need to consider the effect of structural symmetry.

Link prediction limit

Now we derive theoretical formulas for link prediction limit of networks generated from $\mathbf{Q}$. As is stated in the main text, each link's score assigned by a TBPA is exactly equal or proportional to q_{ij}. Therefore, one can simply numerically quantify the TBPA performance entropy H_{TBPA} by ranking the links based on the rankings of their q_{ij} values in $\mathbf{Q}$. One should note that during the ranking of q_{ij} values one should exclude the existing links in the ranking process, because link prediction algorithms only measure the likelihood of unobserved pairs of nodes. Similarly for an artificial network generated from a static random matrix $\mathbf{Q}$, if one simply identifies the ranking of every existing link in the list of all q_{ij}, after excluding the q_{ij} values of other existing links. The obtained ranking distribution of existing links is indeed a sample from the distribution of all q_{ij} within $\mathbf{Q}$ (see Supplementary Figure 13). And the entropy of such ranking distribution should be equivalent to that of the distribution of $\mathbf{Q}$,

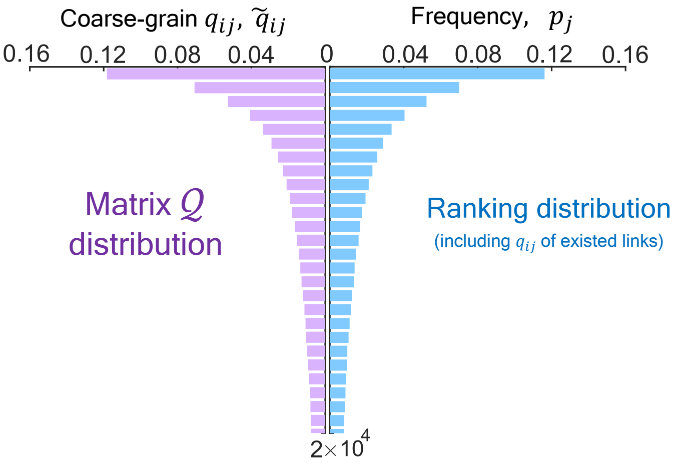

Supplementary Figure 13. Comparison between the ranking distribution and that from random matrix $\mathbf{Q}$ based on E. Coli network. The left part presents the ranking distribution of coarse-grained probabilities $\tilde{q}_{ij}$, each of which is the average value of every N values of q_{ij} ranked in a descending order. While the right part illustrates the ranking distribution of existing links. Each link's ranking is determined from the ranking list of all q_{ij} within $\mathbf{Q}$ without excluding q_{ij} of existing links. The probability p_j is an average value of 100 simulations.

which is given by

$$\begin{aligned} H_{\mathbf{Q}} &= -\sum_{i>j} \frac{q_{ij}}{\sum_{i>j} q_{ij}} \log \frac{q_{ij}}{\sum_{i>j} q_{ij}} \\ &\approx -\sum_{i>j} \frac{q_{ij}}{\frac{N\langle k\rangle}{2}} \log \frac{q_{ij}}{\frac{N\langle k\rangle}{2}} \\ &= -\frac{2}{N\langle k\rangle} \sum_{i>j} q_{ij} (\log q_{ij} - \log \frac{N\langle k\rangle}{2}) \\ &= -\frac{2}{N\langle k\rangle} \sum_{i>j} q_{ij} \log q_{ij} + \log \frac{N\langle k\rangle}{2} \end{aligned} \tag{27}$$

Recall that in the calculation of algorithm performance entropy, we have divided the ranking range into bins with equal width N. In such case, the distribution of $\mathbf{Q}$ is a coarse-grained version by average every N values of q_{ij} (in the descending order) to give $\tilde{q}_{ij}$. In other words, the number of variable reduces from $\binom{N}{2}$ to $N/2$. Based on the derivation in SI, section VI, one can obtain the relationship between the coarse-grained entropy $\tilde{H}_{\mathbf{Q}}$ and the entropy of $\mathbf{Q}$:

$$\tilde{H}_{\mathbf{Q}} \approx H_{\mathbf{Q}} - \log N \tag{28}$$

In the calculate of H_{TBPA}, we have removed the ranking of existing links, leading to a difference between the

TBPA ranking distribution and the distribution of $\tilde{q}_{ij}$. But such difference contributes negligibly to the calculation of entropy as shown in Fig. 3c in the main text, yielding:

$$H_{\mathrm{TBPA}} \approx \tilde{H}_{\mathbf{Q}} \tag{29}$$

By combining Supplementary Equations27-29, one obtain:

$$H_{\mathrm{TBPA}} \approx -\frac{2}{N\langle k\rangle}\sum_{i>j} q_{ij}\log q_{ij} + \log\frac{\langle k\rangle}{2} \tag{30}$$

We then normalize H_{TBPA} by $\log N - 1$ through the same way of Eqn. 2 in the main text, yielding:

$$H^*_{\mathrm{TBPA}} = \frac{H_{\mathrm{TBPA}}}{\log N - 1} \tag{31}$$

Theoretical linear relationship

Combining Supplementary Equations25-31, the linear relationship between L^* and H^*_{TBPA} is:

$$H^*_{\mathrm{TBPA}} \approx \frac{2[\binom{N}{2}h(q) - N\log N]}{\langle k\rangle N\log\frac{N}{2}}L^* + \frac{\frac{2\log N}{\langle k\rangle} + \log\frac{\langle k\rangle}{2} - \frac{1}{\ln 2}}{\log\frac{N}{2}}. \tag{32}$$

Now we identify the slope and the intercept of Supplementary Equation 32 in the thermodynamic limit that network size N tends to infinity. Let ϕ and η denote the slope and the intercept of Supplementary Equation 32 respectively, i.e.,:

$$\begin{aligned} \phi &= \frac{2[\binom{N}{2}h(p) - N\log N]}{\langle k\rangle N\log\frac{N}{2}}, \\ \eta &= \frac{\frac{2\log N}{\langle k\rangle} + \log\frac{\langle k\rangle}{2} - \frac{1}{\ln 2}}{\log\frac{N}{2}} \end{aligned} \tag{33}$$

When the network size N tends to infinity and $\langle k\rangle$ is finite, the limiting slope value is estimated as:

$$\begin{aligned}
\lim_{N\to\infty}\phi &= \frac{2[\binom{N}{2}h(p)-N\mathrm{log}N]}{\langle k\rangle N\mathrm{log}\frac{N}{2}} \\
&\approx \frac{N^2[-p\mathrm{log}p-(1-p)\mathrm{log}(1-p)]-2N\mathrm{log}N}{\langle k\rangle N\mathrm{log}N} \\
&= \frac{N[-p\mathrm{log}p-(1-p)\mathrm{log}(1-p)]-2\mathrm{log}N}{\langle k\rangle\mathrm{log}N} \\
&\approx \frac{N[-\frac{\langle k\rangle}{N}\mathrm{log}\frac{\langle k\rangle}{N}-(1-\frac{\langle k\rangle}{N})\mathrm{log}(1-\frac{\langle k\rangle}{N})]-2\mathrm{log}N}{\langle k\rangle\mathrm{log}N}-\frac{2}{\langle k\rangle} \\
&= \frac{-\langle k\rangle\mathrm{log}\frac{\langle k\rangle}{N}-(N-\langle k\rangle)\mathrm{log}(1-\frac{\langle k\rangle}{N})}{\langle k\rangle\mathrm{log}N}-\frac{2}{\langle k\rangle} \\
&\approx -\frac{\mathrm{log}\frac{\langle k\rangle}{N}}{\mathrm{log}N}-\frac{2}{\langle k\rangle} \\
&= 1-\frac{2}{\langle k\rangle}-\frac{\mathrm{log}\langle k\rangle}{\mathrm{log}N}
\end{aligned} \tag{34}$$

Similarly, the limiting intercept value is estimated as:

$$\begin{aligned}
\lim_{N\to\infty}\eta &= \frac{\frac{2\mathrm{log}N}{\langle k\rangle}+\mathrm{log}\frac{\langle k\rangle}{2}-\frac{1}{\mathrm{ln}2}}{\mathrm{log}\frac{N}{2}} \\
&\approx \frac{\frac{2\mathrm{log}N}{\langle k\rangle}+\mathrm{log}\langle k\rangle}{\mathrm{log}N} \\
&= \frac{2}{\langle k\rangle}+\frac{\mathrm{log}\langle k\rangle}{\mathrm{log}N}
\end{aligned} \tag{35}$$

When the network size N is large, Supplementary Equation 32 simplifies to:

$$H^*_{\mathrm{TBPA}} \approx (1-\frac{2}{\langle k\rangle}-\frac{\mathrm{log}\langle k\rangle}{\mathrm{log}N})L^*+\frac{2}{\langle k\rangle}+\frac{\mathrm{log}\langle k\rangle}{\mathrm{log}N}. \tag{36}$$

Supplementary Figure 14 presents how the value of $1-\frac{2}{\langle k\rangle}-\frac{\mathrm{log}\langle k\rangle}{\mathrm{log}N}$ changes with network size N in different mean degree $\langle k\rangle$.

Note that Supplementary Equation 36 can be further approximated in the thermodynamic limit where $\mathrm{log}N \gg \mathrm{log}\langle k\rangle$:

$$H^*_{\mathrm{TBPA}} \approx (1-\frac{2}{\langle k\rangle})L^*+\frac{2}{\langle k\rangle}. \tag{37}$$

In fact, as we can see in Supplementary Figure 14, the approximation result in Supplementary Equation 37 is only accurate in the artificial network with size larger than 10^{100}.

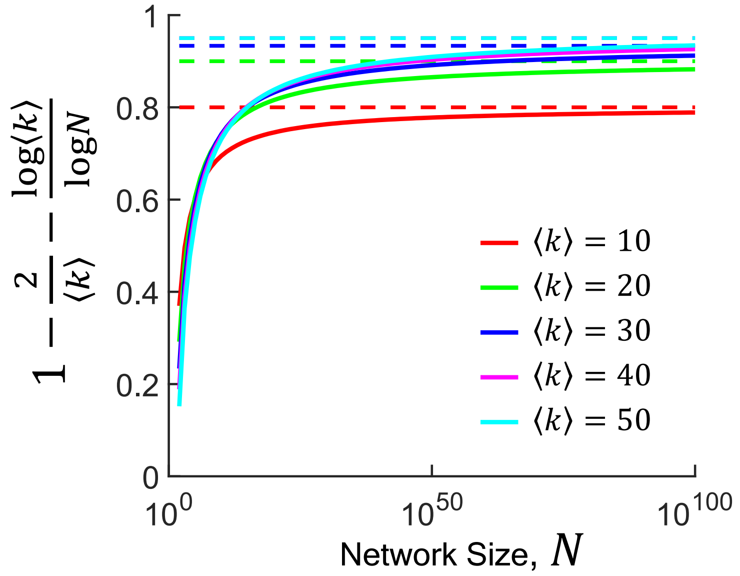

Supplementary Figure 14. Values of the slope in Supplementary Equation 36 vs. network size N with various mean degree $\langle k\rangle$. The dash line represents the thermodynamic limit slope in Supplementary Equation 37.

Theoretical linear relationship without binning

In this subsection we discuss the linear relationship between the link prediction limit and the shortest compression length when no binning of the link rankings are carried out. In such no-bin case, the TBPA performance entropy $H_{\text{TBPA}}^{\text{nobin}}$ is approximately equal to the entropy of static random matrix $\mathbf{Q}$, i.e.,:

$$\begin{aligned} H_{\text{TBPA}}^{\text{nobin}} &\approx H_{\mathbf{Q}} \\ &\approx -\frac{2}{N\langle k\rangle}\sum_{i>j} q_{ij}\log q_{ij} + \log\frac{N\langle k\rangle}{2} \end{aligned} \tag{38}$$

Similarly, we denote $H_{\text{TBPA}}^{\text{nobin}^*}$ to be the normalized H_{TBPA}^{nobin}, i.e.,:

$$H_{\text{TBPA}}^{\text{nobin}^*} = \frac{H_{\text{TBPA}}^{\text{nobin}}}{\log\binom{N}{2}} \tag{39}$$

Combining Supplementary Equations25-39, one can obtain the linear relationship between the $H_{\text{TBPA}}^{\text{nobin}}$ and the L^* :

$$H_{\text{TBPA}}^{\text{nobin}^*} \approx \frac{2[\binom{N}{2}h(q) - N\log N]}{N\langle k\rangle\log\binom{N}{2}}L^* + \frac{\frac{2\log N}{\langle k\rangle} + \log\frac{N\langle k\rangle}{2} - \frac{1}{\ln 2}}{\log\binom{N}{2}} \tag{40}$$

Similarly, Eqn40 can by simplified in thermodynamic limit:

$$H_{\text{TBPA}}^{\text{nobin}^*} = (\frac{1}{2} - \frac{\log\langle k\rangle}{2\log N} - \frac{1}{\langle k\rangle})L^* + \frac{1}{2} + \frac{\log\langle k\rangle}{2\log N} + \frac{1}{\langle k\rangle} \tag{41}$$

When $\log N \gg \log\langle k\rangle$, we have:

$$H_{\text{TBPA}}^{\text{nobin}^*} = (\frac{1}{2} - \frac{1}{\langle k\rangle})L^* + \frac{1}{2} + \frac{1}{\langle k\rangle} \tag{42}$$

Further validation of theoretical linear relationship on additional edge-independent synthetic networks

In this subsection, we implement two other edge-independent synthetic network models to validate the theoretical linear relationship. The first degree-correlated stochastic block model [29] is a generative model for community structures. According to Ref.[29], node i and j has probability $\theta_i\theta_j\omega_{g_ig_j}$ of being connected, where ω_{rs} is a symmetric matrix of parameters controlling edges between groups r and s, g_i is the group assignment of vertex i, and θ_i is set of parameters controlling the expected degrees of vertices i. We assume the number of communities is 2, apply degree distributions from empirical networks, and use Eqns. 12, 18 and 27 in [29] to determine the values of ω, θ, g. With this we generated an artificial network with 2 communities and same degree distribution as the empirical networks; the community strength can be controlled with parameter λ: when $\lambda = 1$ the links are only within each community; when $\lambda = 0$ the links are random but degrees are preserved similar to our treatment in the main text. After determining the values of ω, θ, g, we obtain the adjacency matrix $\mathbf{Q}$, and use the method in our work to calculate L^* and H^*_{TBPA}. The results are shown in Supplementary Figure 15a. We observe that the degree-corrected stochastic block model still falls on the theoretical linear relationship that is different from the empirical one. In addition, as λ decreases, the community structure weakens, the position of (L^*, H^*_{TBPA}) in the figure shifts upwards, in line with our theoretical results.

For the second latent-geometric network model [30, 31], link probability depends on the latent geometric distance between the two nodes. Here we consider the latent geometric space as 1-D real axis $\mathcal{R}^1$, and the nodes are randomly distributed on the line. According to [30], q_{ij} equals to $(k_ik_j)/2m\delta(i,j)$, where k_i, k_j are the degrees of the nodes, m is the total number of edges, and $\delta(i,j)$ is the edge function. For $\delta(i,j)$, without loss of generality, we consider the following case: the probability of connecting two nodes is inversely related to their distance. Hence, we employ the method in [31], and set $\delta(i,j) = \beta e^{\frac{-d(i,j)}{\alpha L}}$, where $d(i,j)$ is the distance between i and j, and L is the maximum distance between two nodes, $\alpha \in (0,1]$ controls the strength of the geometric influence: larger α

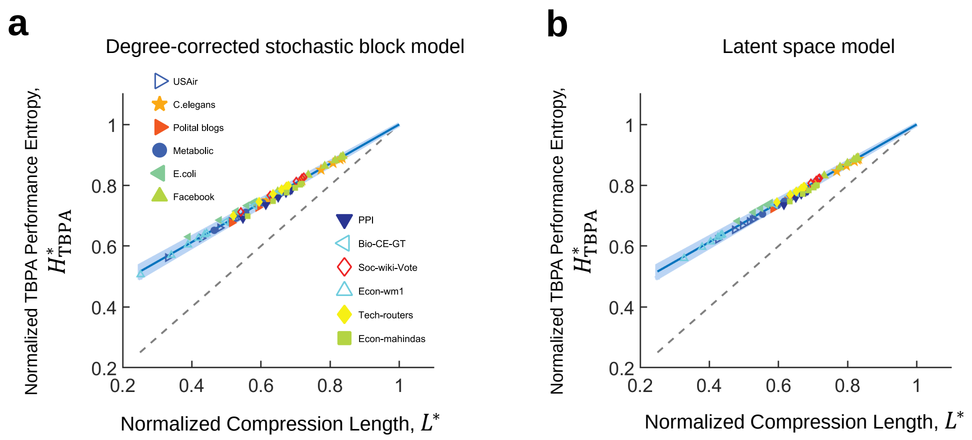

Supplementary Figure 15. Normalized TBPA performance entropy vs. compression length for **a** degree-correlated stochastic block model and **b** latent space model. Both follow the same linear relationship as our theoretical result.

leads to more random connections. While parameter β controls the average degree of the network, ensuring the artificial network is similar in total edges. Next, we use the adjacency matrix $\mathbf{Q}$ to calculate L^* and H^*_{TBPA}. The results are shown in Supplementary Figure 15b. Again, the values fall on the theoretical linear relationship, and the point (L^*, H^*_{TBPA}) shifts towards top right as the geometric influence weakens.

In our theoretical derivation of the linear relationship, both L^* and H^* are based on the matrix $\mathbf{Q}$ that generates the artificial networks, with element q_{ij} denoting the probability of connecting node i and j. Importantly, we did not enforce any particular form of $\mathbf{Q}$. Hence, for the above two models, although they have more complex and different $\mathbf{Q}$s, the predictability properties are still within the analytical scope of our theory. Specifically, as long as the edges are independently formed and their probabilities can be described using adjacency matrix $\mathbf{Q}$, the network's structure predictability-compression length relation should follow our theoretical results in Eqn. 7 in the main text.

Supplementary Note 10: Bounds of prediction precision

In this section, we present a detailed analysis for the upper and lower bounds of prediction precision given the normalized BPAA performance entropy H^*_{BPAA} of a network. Here we take an empirical network USAir as an

example, whose BPAA is RA algorithm [27].

To quantify the upper bound of prediction precision, here we make use of the principle of maximum entropy [12, 32]. Ref.[32] discussed this topic in some details. Particularly, an analytical method was developed to identify the maximum entropy for a given general discrete distribution $\{p_j\}$, which contains T variables $p_1, p_2, ..., p_T$. Based on that, we come by the following lemma:

Lemma 2[32]: Consider the following entropy optimization problem:

$$\max \ z = -\sum_{j=1}^{T} p_j \log p_j$$

$$s.t. \quad \sum_{j=1}^{T} p_j = A$$

where A is a constant $(0 \le A \le 1)$. The distribution that maximizes the entropy z is the uniform distribution $p_1 = p_2 = \cdots = p_T = \frac{A}{T}$, and the corresponding maximum value of z is:

$$z_{\max} = A\log\frac{T}{A} \tag{43}$$

Besides, to quantify *the lower bound* of prediction precision, we need to use the following lemma

Lemma 3: Consider the following entropy optimization problem:

$$\begin{aligned} &\min \ z = -\sum_{j=1}^{T} p_j \log p_j \\ &s.t. \quad \begin{cases} \sum_{j=1}^{T} p_j = A \\ 0 \le p_j \le B, \qquad 1 \le j \le T \end{cases} \end{aligned} \tag{44}$$

where A, B are both constants ($B \neq 0$). The distribution that minimizes the entropy z is as follows:

$$p_j = \begin{cases} B, & j \le \lfloor \frac{A}{B} \rfloor. \\ A - B\lfloor \frac{A}{B} \rfloor, & j = 1 + \lfloor \frac{A}{B} \rfloor. \\ 0, & j \ge 1 + \lfloor \frac{A}{B} \rfloor. \end{cases} \tag{45}$$

where $\lfloor x \rfloor$ means that x is rounded down to the nearest integer. The corresponding minimum value of z is:

$$z_{\min} = -\left\lfloor \frac{A}{B} \right\rfloor B\log B - (A - \left\lfloor \frac{A}{B} \right\rfloor B)\log(A - \left\lfloor \frac{A}{B} \right\rfloor B) \tag{46}$$

Now we prove *Lemma 3* in detail. We construct the Lagrange function [33] L_1 defined by:

$$L_1 = -\sum_{j=1}^{T} p_j \log p_j + \lambda(\sum_{j=1}^{T} p_j - A) + \sum_{j=1}^{T} \alpha_j (p_j - B) - \sum_{j=1}^{T} \beta_j p_j \tag{47}$$

where λ, α_j and $\beta_j (1 \leq j \leq T)$ are Lagrange multipliers according to multiple constraints of the optimization problem. Note that the stationary point for the Lagrange function L_1 satisfies:

$$\frac{\partial L_1}{\partial p_j} = -\mathrm{log} p_j - 1 + \lambda + \alpha_j - \beta_j = 0, \quad 1 \leq j \leq T \tag{48}$$

Besides, according to the Karush-Kuhn-Tucker (KKT) conditions [34] which are the necessary conditions for optimal solutions of nonlinear programming, the stationary point satisfies:

$$\alpha_j (p_j - B) = 0, \quad 1 \leq j \leq T \tag{49}$$

and

$$\beta_j p_j = 0, \quad 1 \leq j \leq T \tag{50}$$

Based on Supplementary Equation 48, p_j can be presented as:

$$p_j = G 2^{\alpha_j - \beta_j}, \quad 1 \leq j \leq T \tag{51}$$

where G is a constant. Moreover, we can see from Supplementary Equations 49,50 that, the value of p_j satisfying the stationary point is one of the following three cases: (i) $p_j = B$ with corresponding $\beta_j = 0$; (ii) $p_j = G$ with $\alpha_j = \beta_j = 0$; (iii) $p_j = 0$ with $\alpha_j = 0$. For convenience, here we denote m_1, m_2, m_3 to be the numbers of p_j satisfying above three cases respectively, yielding:

$$m_1 + m_2 + m_3 = T \tag{52}$$

Because of the constraint $\sum_{j=1}^{T} p_j = A$, we have:

$$m_1 B + m_2 G = A \Rightarrow G = \frac{A - m_1 B}{m_2} \tag{53}$$

Then the entropy z can be represented as:

$$z = -m_1 B \mathrm{log} B - (A - m_1 B) \mathrm{log} \frac{A - m_1 B}{T - m_1 - m_3} \tag{54}$$

Noticeably, the partial derivative of z with respect to m_1 is :

$$\begin{aligned} \frac{\partial z}{\partial m_1} &= -B \mathrm{log} B + B \mathrm{log} \frac{A - m_1 B}{T - m_1 - m_3} + \mathrm{log} e (B - \frac{A - m_1 B}{T - m_1 - m_3}) \\ &= -B \mathrm{log} \frac{B}{G} + \mathrm{log} e (B - G) \\ &= \mathrm{log} e (-B \mathrm{ln} \frac{B}{G} + B - G) \end{aligned} \tag{55}$$

By substituting $\frac{B}{G}$ for a new variable υ, we can easily obtain the inequality $\upsilon \ln \upsilon \geq \upsilon - 1$, which yields

$$\frac{\partial z}{\partial m_1} \leq 0 \tag{56}$$

Similarly, the partial derivative with respect to m_3 is:

$$\frac{\partial z}{\partial m_3} = \frac{\log e (m_1 B - A)}{m_3} < 0 \tag{57}$$

Therefore, the entropy function z is monotonically decreasing at the directions of both m_1, m_3, implying that the minimum value of z is obtained at the maximum value of m_1, m_3. Since $\sum_{j=1}^{T} p_j = A$, the maximal value of m_1 is $\lfloor \frac{A}{B} \rfloor$, which in the same time results in the maximal number of p_j taking 0. Thus the distribution that minimizes the entropy z is as follows:

$$p_j = \begin{cases} B, & j \leq \lfloor \frac{A}{B} \rfloor. \\ A - B \lfloor \frac{A}{B} \rfloor, & j = 1 + \lfloor \frac{A}{B} \rfloor. \\ 0, & j \geq 1 + \lfloor \frac{A}{B} \rfloor. \end{cases} \tag{58}$$

The corresponding minimum value of z is

$$z_{\min} = -\left\lfloor \frac{A}{B} \right\rfloor B \log B - (A - \left\lfloor \frac{A}{B} \right\rfloor B) \log (A - \left\lfloor \frac{A}{B} \right\rfloor B) \tag{59}$$

Upper bound of p_1

Next we derive the upper and lower bound for the prediction precision. In this subsection we prove that, given the normalized BPAA performance entropy H^*_{BPAA} of a network, the upper bound $\overline{p}_1$ of probability p_1 that the missing link is ranked in the top N positions (the first bin) satisfies:

$$-\overline{p}_1 \log \overline{p}_1 - (1 - \overline{p}_1) \log (\frac{1 - \overline{p}_1}{N/2 - 1}) = (\log N - 1) H^*_{\mathrm{BPAA}} \tag{60}$$

As described in the main text and Supplementary Note 6, we measure the ranking distribution by dividing the ranking into $N/2$ bins with equal-width N and calculating the entropy based on the probability distribution $\{p_j\}$ of these $N/2$ bins. Noticeably, we observe that the ranking distributions obtained by all of the prediction algorithms we used almost satisfy:

$$p_1 \geq p_2 \geq \ldots \geq p_{N/2} \tag{61}$$

By taking Supplementary Equation 61 and the given H^*_{BPAA} value into consideration, the solution of the upper

bound $\overline{p}_1$ of p_1 can be reduced to the following optimization problem:

$$\begin{aligned} \max \quad & p_1 \\ s.t. \quad & \begin{cases} p_1 \geq p_2 \geq \dots \geq p_{N/2} \\ -\sum_{j=1}^{N/2} p_j \mathrm{log} p_j = (\mathrm{log} N - 1) H^*_{\mathrm{BPAA}} \\ \sum_{j=1}^{N/2} p_j = 1 \end{cases} \end{aligned} \tag{62}$$

It is infeasible to solve this problem directly due to the second constraint which is non-linear. Therefore, we define $H(\{p_j\})$ to be the entropy function of a given distribution $\{p_j\}$ and then identify the maximum value of $H(\{p_j\})$ when the probability p_1 is given. Note that $H(\{p_j\})$ can be represented as:

$$H(\{p_j\}) = -p_1 \mathrm{log} p_1 - \sum_{j=2}^{N/2} p_j \mathrm{log} p_j \tag{63}$$

When p_1 is given, it is obvious that maximizing the value of $H(\{p_j\})$ is equivalent to maximize the second term of the right-hand side of Supplementary Equation 63, i.e., $-\sum_{j=2}^{N/2} p_j \mathrm{log} p_j$. Note that

$$\sum_{j=2}^{N/2} p_j = 1 - p_1 \tag{64}$$

According to *Lemma 2*, the distribution that maximizes $-\sum_{j=2}^{N/2} p_j \mathrm{log} p_j$ is the uniform distribution, i.e., $p_2 = p_3 = \dots = p_{N/2} = \frac{1-p_1}{N/2-1}$, and the corresponding maximum value of $-\sum_{j=2}^{N/2} p_j \mathrm{log} p_j$ is $-(1-p_1)\mathrm{log}(\frac{1-p_1}{N/2-1})$. Let $\overline{H}(p_1)$ denotes the maximum value of $H(\{p_j\})$ given p_1, that is:

$$\overline{H}(p_1) = -p_1 \mathrm{log} p_1 - (1-p_1)\mathrm{log}(\frac{1-p_1}{N/2-1}) \tag{65}$$

Supplementary Figure 16 shows the optimal distribution $\{p_j^*\}$ whose entropy is $\overline{H}(p_1)$. Because of the constrain $p_1 \geq p_2 \geq \dots \geq p_{N/2}$, we can see that p_1 is the maximum value among $\{p_j^*\}$. Since there are $\frac{N}{2}$ variables in $\{p_j^*\}$, the minimum value p_1 can be take is $\frac{1}{N/2} = \frac{2}{N}$, resulting in the following range of p_1:

$$\frac{2}{N} \leq p_1 \leq 1 \tag{66}$$

To determine the optimal solution of original problem described by Supplementary Equation 62 via $\overline{H}(p_1)$, we need to prove the monotonic decreasing of $\overline{H}(p_1)$ in the range of $[\frac{2}{N}, 1]$. The derivative of $\overline{H}(p_1)$ is:

$$\begin{aligned} \frac{\partial \overline{H}(p_1)}{\partial p_1} &= \frac{\partial[-p_1 \mathrm{log} p_1 - (1-p_1)\mathrm{log}(\frac{1-p_1}{N/2-1})]}{\partial p_1} \\ &= -(\mathrm{log} p_1 + 1) + \mathrm{log}(\frac{1-p_1}{N/2-1}) + 1 \\ &= \mathrm{log}(\frac{1-p_1}{N/2-1}) - \mathrm{log} p_1 \end{aligned} \tag{67}$$

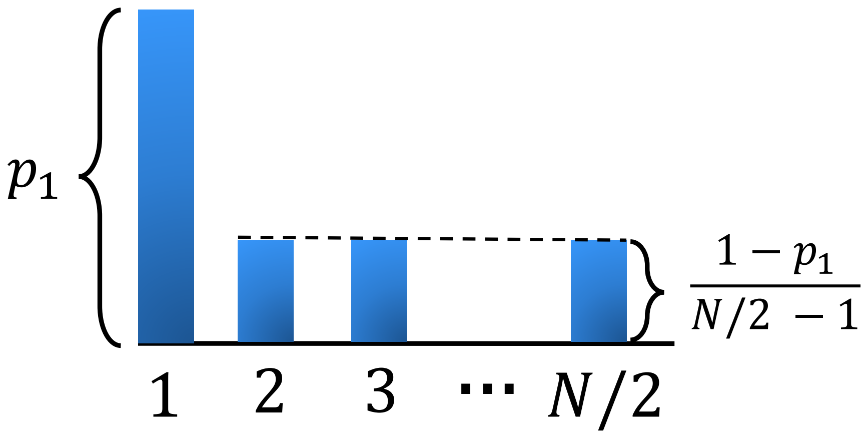

Supplementary Figure 16. Illustration of the optimal distribution $\{p_j^*\}$ that obtains $\overline{H}(p_1)$.

Note that $p_1 \geq \frac{2}{N}$, which yields $\frac{1-p_1}{N/2-1} - p_1 \leq 0$, then we obtain $\frac{\partial \overline{H}(p_1)}{\partial (p_1)} \leq 0$. In other words, $\overline{H}(p_1)$ is monotonically decreasing in the range $[\frac{2}{N}, 1]$.

Due to the fact that $\overline{H}(p_1)$ is monotonically decreasing as well as $\overline{H}(1) = 0$, there must exist a point of intersection for $\overline{H}(p_1)$ and the given $H = H^*_{\mathrm{BPAA}} \cdot (\log N - 1)$ in the range $[2/N, 1]$. The p_1 obtained from the intersection point is exactly the optimal solution $\overline{p}_1$ of the original problem. Overall, the upper bound $\overline{p}_1$ of p_1 can be obtained by solving the following equation (also shown in Supplementary Figure 17):

$$-\overline{p}_1 \log \overline{p}_1 - (1-\overline{p}_1)\log(\frac{1-\overline{p}_1}{N/2-1}) = (\log N - 1)H^*_{\mathrm{BPAA}} \tag{68}$$

Lower bound of p_1

In this subsection we prove that, given the normalized BPAA performance entropy H^*_{BPAA} of a network and the constraint $p_1 \geq p_2 \geq ... \geq p_{N/2}$, the lower bound $\underline{p}_1$ of probability p_1 can be approximated as:

$$\underline{p}_1 \approx 2^{-(\log N - 1)H^*_{\mathrm{BPAA}}} \tag{69}$$

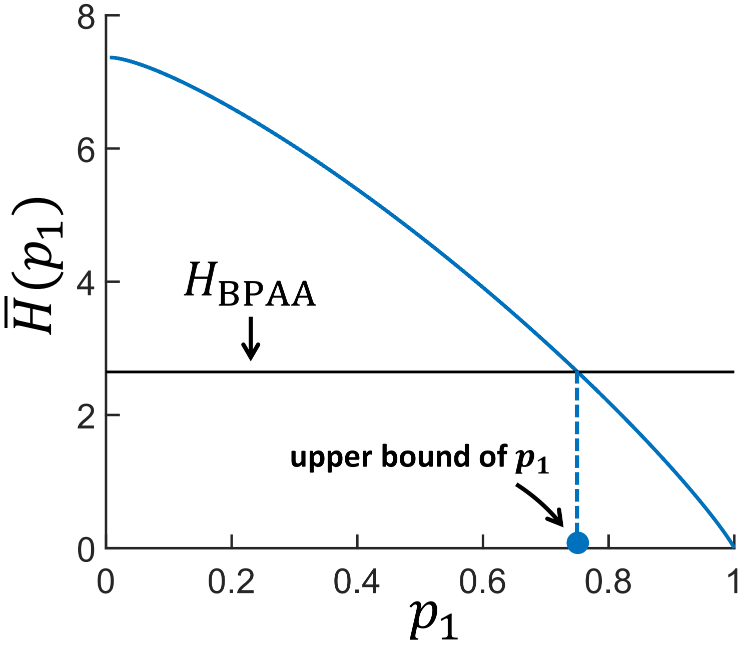

Supplementary Figure 17. Determination of the upper bound $\overline{p}_1$ in USAir network.

As before, here we reduce the solution of the lower bound $\underline{p}_1$ to the following optimization problem

$$\min \quad p_1$$
$$s.t. \begin{cases} p_1 \geq p_2 \geq ... \geq p_{N/2} \\ -\sum_{j=1}^{N/2} p_j \log p_j = (\log N - 1) H^*_{\text{BPAA}} \\ \sum_{j=1}^{N/2} p_j = 1 \end{cases}$$

Similarly, we identify the minimum value of $H(\{p_j\})$ when the probability p_1 is given. Based on *Lemma2*, the minimum value of $H(\{p_j\})$ with given p_1 is:

$$\underline{H}(p_1) = -\left\lfloor \frac{1}{p_1} \right\rfloor p_1 \log p_1 - (1 - \left\lfloor \frac{1}{p_1} \right\rfloor p_1) \log(1 - \left\lfloor \frac{1}{p_1} \right\rfloor p_1) \tag{70}$$

The resulting optimal distribution $\{p^*_j\}$ (see Supplementary Figure 18) is:

$$p^*_j = \begin{cases} p_1, & j \leq \lfloor \frac{1}{p_1} \rfloor. \\ 1 - p_1 \lfloor \frac{1}{p_1} \rfloor, & j = 1 + \lfloor \frac{1}{p_1} \rfloor. \\ 0, & j \geq 1 + \lfloor \frac{1}{p_1} \rfloor. \end{cases} \tag{71}$$

Noticeably, although the inequality constraint of *Lemma2* ($p_j \leq p_1$) is different from that of the original problem ($p_1 \geq p_2 \geq ... \geq p_{N/2}$), we find that the optimal distribution $\{p^*_j\}$ obtained by *Lemma2* still satisfies $p_1 \geq p_2 \geq ... \geq p_{N/2}$.

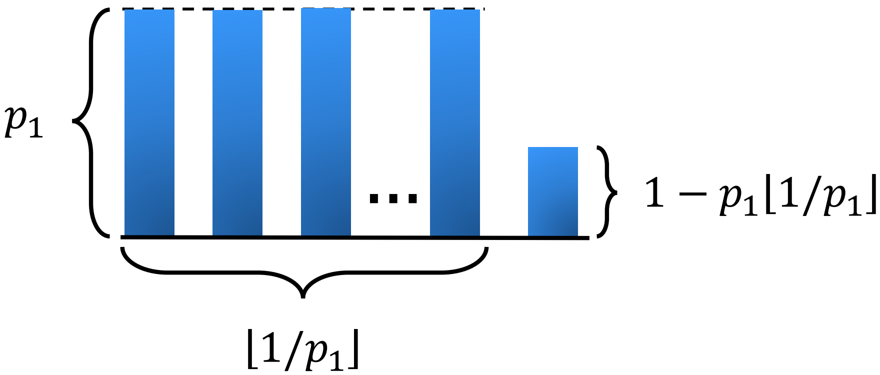

Supplementary Figure 18. Illustration of the optimal distribution $\{p_j^*\}$ that obtains $\underline{H}(p_1)$.

Further, $\underline{H}(p_1)$ can be approximated in the case of small p_1, where we can reasonably assume that p_1 is exactly divisible by 1, yielding $1 - \left\lfloor \frac{1}{p_1} \right\rfloor p_1 = 0$. Therefore, using Supplementary Equation 70, the approximation $\underline{H}(p_1)$ is given by:

$$\hat{\underline{H}}(p_1) = -\text{log}p_1 \tag{72}$$

Supplementary Figure 19 shows the value of $\hat{\underline{H}}(p_1)$ and $\underline{H}(p_1)$ vs p_1. We can see that there is almost no difference between $\hat{\underline{H}}(p_1)$ and $\underline{H}(p_1)$ when p_1 is small.

Since $\frac{\partial \hat{\underline{H}}(p_1)}{\partial p_1} = -\frac{1}{p_1} \leq 0$, $\hat{\underline{H}}(p_1)$ is also monotonically decreasing with respect of p_1 in the range of $[2/N, 1]$. Also note that we have $\hat{\underline{H}}(1) = 0$. As a result, given the BPAA performance entropy $H_{BPAA} = (\text{log}N - 1)H^*_{\text{BPAA}}$, there must exists an intersection point for H_{BPAA} and $\hat{\underline{H}}(p_1)$. The p_1 obtained from this point is exactly the lower bound $\underline{p}_1$ of p_1, i.e.:

$$\begin{aligned} -\text{log}\underline{p}_1 &= (\text{log}N - 1)H^*_{\text{BPAA}} \\ \Rightarrow \underline{p}_1 &= 2^{-(\text{log}N-1)H^*_{\text{BPAA}}} \end{aligned} \tag{73}$$

In the case that p_1 is relatively large, there exists a difference between the approximation $\hat{\underline{H}}(p_1)$ and the real value $\underline{H}(p_1)$. In this sense we should use $\underline{H}(p_1)$ rather than $\hat{\underline{H}}(p_1)$ to determine $\underline{p}_1$. In other words, the lower bound $\underline{p}_1$ of p_1 satisfies:

$$-\left\lfloor \frac{1}{\underline{p}_1} \right\rfloor \underline{p}_1 \text{log}\underline{p}_1 - (1 - \left\lfloor \frac{1}{\underline{p}_1} \right\rfloor \underline{p}_1)\text{log}(1 - \left\lfloor \frac{1}{\underline{p}_1} \right\rfloor \underline{p}_1) = (\text{log}N - 1)H^*_{\text{BPAA}} \tag{74}$$

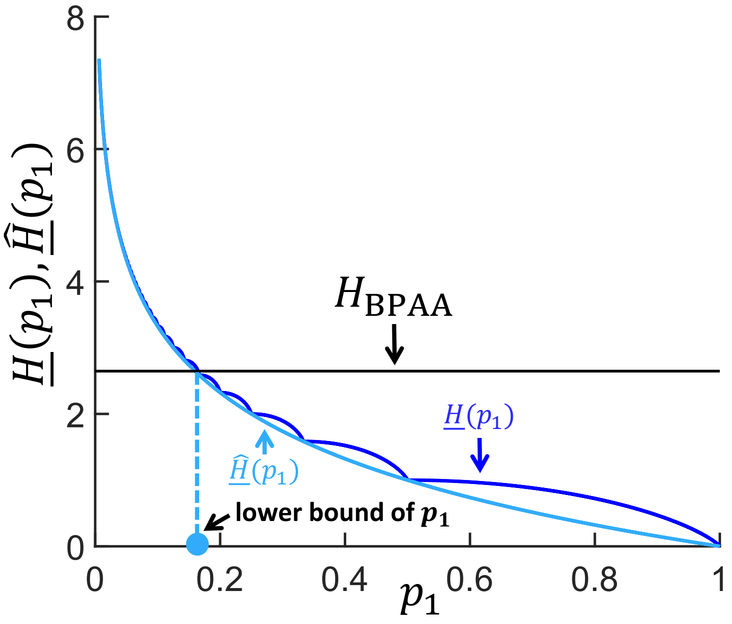

Supplementary Figure 19. Determination of the lower bound $\underline{p}_1$ in USAir network.

Upper bound of $p_1 + p_2 + ... + p_C$

Furthermore, we can determine the upper and lower bound for the precision of missing links in the first C intervals, i.e., the upper and lower bound of the sum $p_1 + p_2 + ... + p_C$. For convenience, in the main text we define P_C to be the probability of the presence of missing links in the top CN positions in the ranking:

$$P_C = \sum_{j=1}^{C} p_j \tag{75}$$

Now we prove that, given the normalized algorithm performance entropy H^* of a network and the constraint $p_1 \geq p_2 \geq ... \geq p_{N/2}$, the upper bound $\overline{P}_C$ of P_C satisfies:

$$\begin{cases} -\overline{P}_C \log\frac{\overline{P}_C}{C} - (1-\overline{P}_C)\log(\frac{1-\overline{P}_C}{N/2-C}) = (\log N - 1)H^*_{\rm BPAA}, & H^*_{\rm BPAA} \geq \frac{\log C}{\log N - 1} \\ \overline{P}_C = 1, & H^*_{\rm BPAA} < \frac{\log C}{\log N - 1} \end{cases} \tag{76}$$

Similarity, we consider the following optimization problem:

$$\begin{aligned} \max \quad & P_C = \sum_{j=1}^{C} p_j \\ s.t. \quad & \begin{cases} p_1 \geq p_2 \geq ... \geq p_{N/2} \\ -\sum_{j=1}^{N/2} p_j \log p_j = (\log N - 1)H^*_{\rm BPAA} \\ \sum_{j=1}^{N/2} p_j = 1 \end{cases} \end{aligned} \tag{77}$$

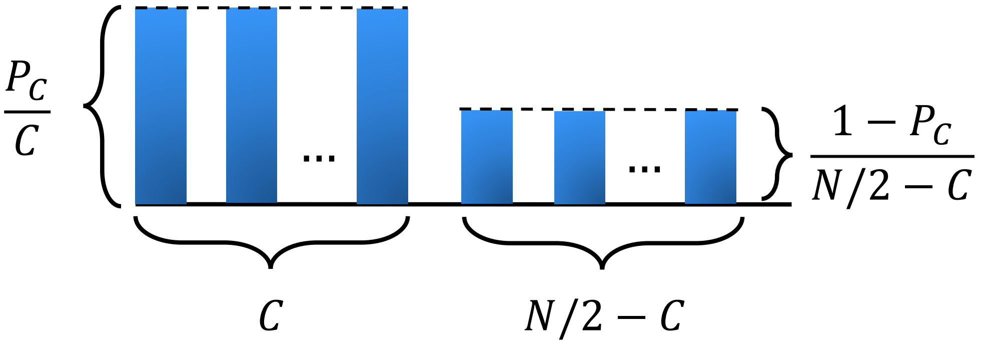

Supplementary Figure 20. Illustration of the optimal distribution $\{p_j^*\}$ that obtains $\overline{H}(P_C)$.

Note that the entropy function $H(\{p_j\})$ can be rewritten as:

$$H(\{p_j\}) = -\sum_{j=1}^{C} p_j \mathrm{log} p_j - \sum_{j=C+1}^{N/2} p_j \mathrm{log} p_j \tag{78}$$

where $\sum_{j=1}^{C} p_j = P_C$. Due to the constraint $p_1 \geq p_2 \geq ... \geq p_{N/2}$, the range of P_C is:

$$\frac{2C}{N} \leq P_C \leq 1 \tag{79}$$

Now we begin to determine the maximum value of $H(\{p_j\})$ when P_C is given. According to Supplementary Equation 78, we partition the entropy $H(\{p_j\})$ into two parts: the entropy of probabilities $p_1, p_2, ..., p_C$ satisfying $p_1 + p_2 + ... + p_C = P_C$; and the remained entropy of $p_{C+1}, p_{C+2}, ..., p_{N/2}$ satisfying $p_{C+1} + p_{C+2} + ... + p_{N/2} = 1 - P_C$. By utilizing *Lemma1*, we can easily come to the conclusion that both of the distributions that maximize the entropies are the uniform distributions. Overall, the optimal distribution is (shown in Supplementary Figure 20)

$$p_j^* = \begin{cases} \frac{P_C}{C}, & 1 \leq j \leq C. \\ \frac{1-P_C}{N/2-C}, & C < j \leq \frac{N}{2}. \end{cases} \tag{80}$$

The corresponding maximum value of $H(\{p_j\})$ given P_C is:

$$\overline{H}(P_C) = -P_C \mathrm{log}(\frac{P_C}{C}) - (1 - P_C)\mathrm{log}(\frac{1 - P_C}{N/2 - C}) \tag{81}$$

Since $P_C \geq \frac{2C}{N}$, we have $\frac{P_C}{C} \geq \frac{1-P_C}{N/2-C}$. Therefore Supplementary Equation 80 satisfies the constrain $p_1 \geq p_2 \geq ... \geq p_{N/2}$.

To determine the optimal solution of original problem described by Supplementary Equation 77 via $\overline{H}(P_C)$, we

need to prove the monotonic decreasing of $\overline{H}(P_C)$. The derivative of $\overline{H}(P_C)$ is:

$$\begin{aligned}\frac{\partial\overline{H}(P_C)}{\partial P_C}=&\frac{\mathrm{d}[-P_C\log(\frac{P_C}{C})-(1-P_C)\log(\frac{1-P_C}{N/2-C})]}{\mathrm{d}P_C}\\=&-(\log\frac{P_C}{C}+1)+\log\frac{1-P_C}{N/2-C}+1\\=&\log\frac{1-P_C}{N/2-C}-\log\frac{P_C}{C}\end{aligned}\tag{82}$$

Due to the fact that $\frac{1-P_C}{N/2-C}\leq\frac{P_C}{C}$, resulting in $\log\frac{1-P_C}{N/2-C}-\log\frac{P_C}{C}\leq 0$, we conclude that $\overline{H}(P_C)$ is monotonically decreasing with respect of P_C in $[\frac{2C}{N},1]$.

Noticeably when $P_C=1$, we have $\overline{H}(1)=\log C$, which means there is one or no intersection point for the given BPAA performance entropy $H_{BPAA}=(\log N-1)H^*_{\mathrm{BPAA}}$ and $\overline{H}(P_C)$, depending on the specific value of H_{BPAA}:

- **One intersection point:** When

$$H_{\mathrm{BPAA}}\geq\overline{H}(1)\tag{83}$$

  i.e., $H^*_{\mathrm{BPAA}}\geq\frac{\log C}{\log N-1}$, there must exist an intersection point for H_{BPAA} and $\overline{H}(P_C)$. Similarly to the case of determining $\overline{p}_1$, the P_C obtained from the intersection point is exactly the optimal solution $\overline{P}_C$ of the original problem, as shown in Supplementary Figure 21 ($C=1,2,4$).

- **No intersection point:** On the contrary, when

$$H_{\mathrm{BPAA}}<\overline{H}(1)\tag{84}$$

  i.e.,$H^*_{\mathrm{BPAA}}<\frac{\log C}{\log N-1}$, there is no intersection point between H_{BPAA} and $\overline{H}(P_C)$. In this case we should choose the boundary value as the optimal value of the ordinal problem since each P_C in the range of $[\frac{2C}{N},1]$ is a feasible solution, i.e., $\overline{P}_C=1$ as shown by Supplementary Figure 21 ($C=8$).

Overall, we conclude that the upper bound $\overline{P}_C$ of P_C satisfies:

$$\begin{cases}-\overline{P}_C\log\frac{\overline{P}_C}{C}-(1-\overline{P}_C)\log(\frac{1-\overline{P}_C}{N/2-C})=(\log N-1)H^*_{\mathrm{BPAA}}, & H^*_{\mathrm{BPAA}}\geq\frac{\log C}{\log N-1}\\ \overline{P}_C=1, & H^*_{\mathrm{BPAA}}<\frac{\log C}{\log N-1}\end{cases}\tag{85}$$

Note that the upper bound $\overline{p}_1$ of p_1 is just a special case of P_C when $C=1$.

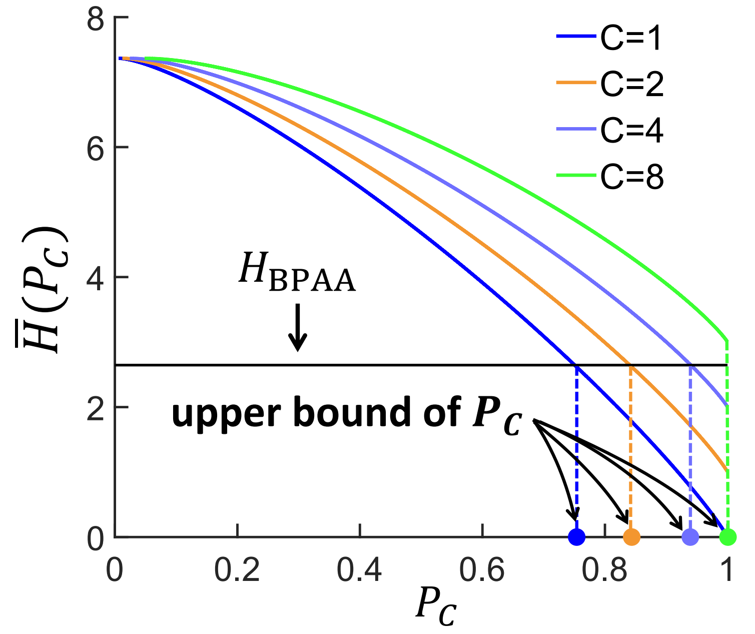

Supplementary Figure 21. Determination of the upper bound $\overline{P}_C$ in USAir network. The horizontal line denotes the specific value of $\overline{P}_C$.

Lower bound of $p_1 + p_2 + ... + p_C$

In this subsection we prove that, given the normalized BPAA performance entropy H^*_{BPAA} of a network and the constraint $p_1 \geq p_2 \geq ... \geq p_{N/2}$, the lower bound $\underline{P_C}$ of P_C satisfies:

$$\underline{P}_C = \min\{P_C : -\gamma(P_C, \epsilon)\log\gamma(P_C, \epsilon) - (1 - \gamma(P_C, \epsilon))\log\epsilon = (\log N - 1)H^*_{\text{BPAA}}\} \tag{86}$$

where ϵ $(0 < \epsilon \leq \frac{P_C}{C})$ denotes the specific value of probability p_C of the Cth interval and $\gamma(P_C, \epsilon) = P_C - (C - 1)\epsilon$.

We start by considering the following optimization problem:

$$\min \quad P_C = \sum_{j=1}^{C} p_j$$

$$s.t. \quad \begin{cases} p_1 \geq p_2 \geq ... \geq p_{N/2} \\ -\sum_{j=1}^{N/2} p_j \log p_j = (\log N - 1)H^*_{\text{BPAA}} \\ \sum_{j=1}^{N/2} p_j = 1 \end{cases}$$

Similarly, we determine the minimum value of $H(\{p_j\})$ when P_C is given. Let us consider the special case of $P_C = 1$, i.e., $p_1 + p_2 + ... + p_C = 1$. Utilizing the fact that the entropy should be 0 when there is no

any uncertainty, it is trivial to show that minimum value of $H(P_C)$ when $P_C = 1$ is 0, with the corresponding distribution $p_1 = 1, p_2 = p_3 = ... = p_C = 0$.

Now we study in detail the case of $P_C \neq 1$, i.e., $\frac{2C}{N} \leq P_C < 1$. To solve the problem efficiently, here we introduce ϵ which represents the specific value of the probability p_C of the Cth interval. That is:

$$p_C = \epsilon, \tag{87}$$

Now we turn to determine the minimum value of $H(\{p_j\})$ given both P_C and ϵ. As we have said, $H(\{p_j\})$ can be represented as $-\sum_{j=1}^{C} p_j \text{log} p_j - \sum_{j=C+1}^{N/2} p_j \text{log} p_j$. For convenience, let H_1, H_2 denotes the entropy functions of $p_1, p_2, ..., p_C$ and $p_{C+1}, p_{C+2}, ..., p_{N/2}$ respectively, i.e.:

$$\begin{aligned} H_1 &= -\sum_{j=1}^{C} p_j \text{log} p_j, \\ H_2 &= -\sum_{j=C+1}^{N/2} p_j \text{log} p_j \end{aligned} \tag{88}$$

Next we identify the minimum value of H_1,H_2 respectively:

- $\underline{H}_1$: With given P_C and ϵ, the optimization problem for the minimum value of H_1 is:

$$\min \quad H_1 = -\sum_{j=1}^{C} p_j \text{log} p_j$$
$$s.t. \quad \begin{cases} p_1 \geq p_2 \geq ... \geq p_C = \epsilon \\ \sum_{j=1}^{C} p_j = P_C \end{cases}$$

  Since ϵ is the smallest probability among p_j $(1 \leq j \leq C)$, p_j can be represented as $p_j = (1 + a_j)\epsilon$, where $a_j \geq 0$. Based on that, H_1 can be rewritten as:

$$\begin{aligned} H_1 &= -\sum_{j=1}^{C} p_j \text{log} p_j \\ &= -\sum_{j=1}^{C} ((1 + a_j)\epsilon) \text{log}((1 + a_j)\epsilon) \\ &= -\epsilon \sum_{j=1}^{C} (1 + a_j)[\text{log}(1 + a_j) + \text{log}\epsilon] \\ &= -\epsilon \text{log}\epsilon \sum_{j=1}^{C} (1 + a_j) - \epsilon \sum_{j=1}^{C} (1 + a_j) \text{log}(1 + a_j) \\ &= -\epsilon \text{log}\epsilon P_C - \epsilon \sum_{j=1}^{C} (1 + a_j) \text{log}(1 + a_j) \end{aligned}$$

Due to the fact that ϵ, P_C are both given constants, it is obvious that minimizing the value of H_1 is equivalent to minimizing -$\sum_{j=1}^{C}(1+a_j)\log(1+a_j)$. To enhance readability, we denote $b_j = 1+a_j, F = \frac{P_C}{\epsilon}$, yielding:

$$\min \quad z = -\sum_{j=1}^{C} b_j \log b_j$$

$$s.t. \quad \begin{cases} \sum_{j=1}^{C} b_j = F, \\ b_j \geq 1, \quad 1 \leq j \leq T \end{cases}$$

Here we employ the method of Lagrange multipliers to solve above problem. We construct the Lagrange function L_2 defined by:

$$L_2 = \sum_{j=1}^{C} b_j \log b_j + \lambda(\sum_{j=1}^{C} b_j - F) - \sum_{j=1}^{C} \alpha_j b_j - 1 \tag{89}$$

where λ, α_j are Lagrange multiplier according to multiple constraints of the optimization problem. The stationary point for the Lagrange function L_2 satisfies:

$$\frac{\partial L_2}{\partial b_j} = -\log b_j - 1 + \lambda + \alpha_j = 0, \quad 1 \leq j \leq T \tag{90}$$

Once again, the KKT conditions [34] imply that the stationary point satisfies:

$$\alpha_j(b_j - 1) = 0, \quad 1 \leq j \leq T \tag{91}$$

Using Supplementary Equation 90, we can represented b_j as:

$$b_j = G2^{-\alpha_j}, \quad 1 \leq j \leq T \tag{92}$$

where G is a constant. Based on Supplementary Equation 91, we can see the value of b_j satisfying the stationary point is one of the following two cases: (i) $b_j = G$ with $\alpha_j = 0$; (ii) $b_j = 1$. Let k_1, k_2 denote the numbers of b_j satisfying above two cases with:

$$k_1 + k_2 = C \tag{93}$$

Because of the constraint $\sum_{j=1}^{C} b_j = F$, we have:

$$k_1 G + k_2 = F \Rightarrow G = \frac{F - k_2}{k_1} \tag{94}$$

Combing Supplementary Equations 89,94, the optimization function z can be written as:

$$z = -(F - k_2)\log\frac{F - k_2}{C - k_2} \tag{95}$$

The derivative of z is:

$$\begin{aligned}\frac{\partial z}{\partial k_2} &= \log\frac{F-k_2}{C-k_2} + \log e(1-\frac{F-k_2}{C-k_2}) \\ &= -\log\frac{1}{G} + \log e(1-G) \\ &= \log e(-\ln\frac{1}{G} + 1 - G)\end{aligned} \tag{96}$$

According the derivation of *Lemma2*, we have $-\ln\frac{1}{G} + 1 - G \le 0$, resulting in $\frac{\partial z}{\partial k_2} \le 0$. Therefore, z is monotonically decreasing with respect to k_2. In other words, the minimum value $z_{\min}$ of z is obtained at the largest k_2. Since $F = \frac{P_C}{\epsilon} \ge C$, the maximum value of k_2 is $C-1$. In summary, we arrive at the optimal distribution $\{b_j^*\}$

$$b_j^* = \begin{cases} F-(C-1), & j=1 \\ 1, & 2 \le j \le C. \end{cases} \tag{97}$$

Therefore, we have:

$$a_j^* = \begin{cases} \frac{P_C}{\epsilon} - C, & j=1 \\ 0, & 2 \le j \le C. \end{cases} \tag{98}$$

Substituting a_j^* into the objective function H_1, the optimal distribution $\{p_j^*\}$ $(1 \le j \le C)$ is given by:

$$p_j^* = \begin{cases} P_C - \epsilon(C-1), & j=1 \\ \epsilon, & 2 \le j \le C. \end{cases} \tag{99}$$

For better readability, let $\gamma(P_C, \epsilon) = P_C - \epsilon(C-1)$. Then the minimum value of H_1 given P_C and ϵ is:

$$\underline{H}_1 = -\gamma(P_C,\epsilon)\log\gamma(P_C,\epsilon) - (C-1)\epsilon\log\epsilon \tag{100}$$

- $\underline{H}_2$: Similarly, we consider the following optimization problem of H_2:

$$\begin{aligned} \min \quad & H_2 = -\sum_{j=C+1}^{N/2} p_j \log p_j \\ s.t. \quad & \begin{cases} \epsilon \ge p_{C+1} \ge \dots \ge p_{N/2} \\ \sum_{j=C+1}^{N/2} p_j = 1 - P_C \end{cases} \end{aligned}$$

Since the maximum possible value of each $p_j (C+1 \le j \le N/2)$ is ϵ, by using Lemma2 we come to the following optimal distribution $\{p_j^*\}$ $(C+1 \le j \le N/2)$:

$$p_j^* = \begin{cases} \epsilon, & C+1 \le j \le C+1+\lfloor\frac{1-P_C}{\epsilon}\rfloor \\ 1-P_C-\epsilon\lfloor\frac{1-P_C}{\epsilon}\rfloor, & j = C+2+\lfloor\frac{1-P_C}{\epsilon}\rfloor \\ 0, & j > C+2+\lfloor\frac{1-P_C}{\epsilon}\rfloor. \end{cases} \tag{101}$$

For better readability, let $\phi(P_C,\epsilon) = \epsilon\lfloor\frac{1-P_C}{\epsilon}\rfloor$. Then the minimum value of H_2 given P_C and ϵ is:

$$\underline{H}_2 = -(1-P_C-\phi(P_C,\epsilon))\log(1-P_C-\phi(P_C,\epsilon)) - \lfloor\frac{1-P_C}{\epsilon}\rfloor\epsilon\log\epsilon \tag{102}$$

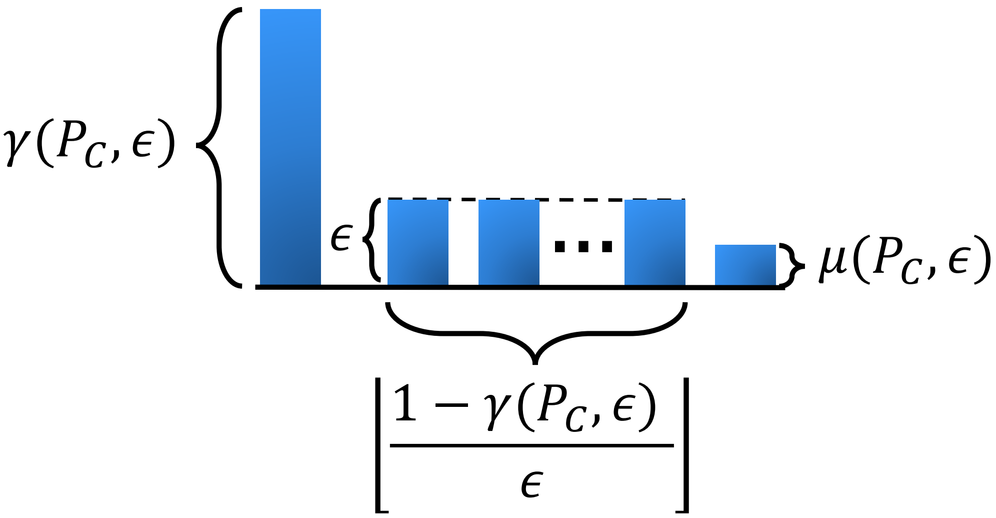

Supplementary Figure 22. Illustration of the optimal distribution $\{p_j^*\}$ that obtains $\underline{H}(P_C)$.

Finally, we let $\mu(P_C,\epsilon) = 1 - \gamma(P_C,\epsilon) - \phi(P_C,\epsilon)$. By combing Supplementary Equations 99,101, we have the following overall optimal distribution $\{p_j^*\}$ $(1 \le j \le N/2)$ (shown in Fig22)

$$p_j^* = \begin{cases} \gamma(P_C,\epsilon), & j = 1 \\ \epsilon, & 2 \le j \le 1 + \lfloor \frac{1-\gamma(P_C,\epsilon)}{\epsilon} \rfloor \\ \mu(P_C,\epsilon), & j = 2 + \lfloor \frac{1-\gamma(P_C,\epsilon)}{\epsilon} \rfloor \\ 0, & j > 2 + \lfloor \frac{1-\gamma(P_C,\epsilon)}{\epsilon} \rfloor. \end{cases} \tag{103}$$

Meanwhile, by combing Supplementary Equations 100,102, the minimum value of $H(\{p_j\})$ given P_C and ϵ is:

$$\underline{H}(P_C,\epsilon) = H_1 + H_2 = -\gamma(P_C,\epsilon)\log\gamma(P_C,\epsilon) - \phi(P_C,\epsilon)\log\epsilon - \mu(P_C,\epsilon)\log\mu(P_C,\epsilon) \tag{104}$$

Now we can numerically determine the minimum value of $H(\{p_j\})$ given P_C by finding the minimum value of $\underline{H}(P_C,\epsilon)$ with fixed P_C and varying ϵ $(0 < \epsilon \le P_C/C)$, i.e.:

$$\underline{H}(P_C) = \min\{\underline{H}(P_C,\epsilon) : 0 < \epsilon \le P_C/C\} \tag{105}$$

Moreover, $\underline{H}(P_C,\epsilon)$ can be approximated in the case of small ϵ, where we can assume that ϵ is exactly divisible by $1-\gamma(P_C,\epsilon)$, yielding $\mu(P_C,\epsilon) = 0$. Therefore, we obtain the approximation $\hat{\underline{H}}(P_C,\epsilon)$ of $\underline{H}(P_C,\epsilon)$:

$$\hat{\underline{H}}(P_C,\epsilon) = -\gamma(P_C,\epsilon)\log\gamma(P_C,\epsilon) - (1-\gamma(P_C,\epsilon))\log\epsilon \tag{106}$$

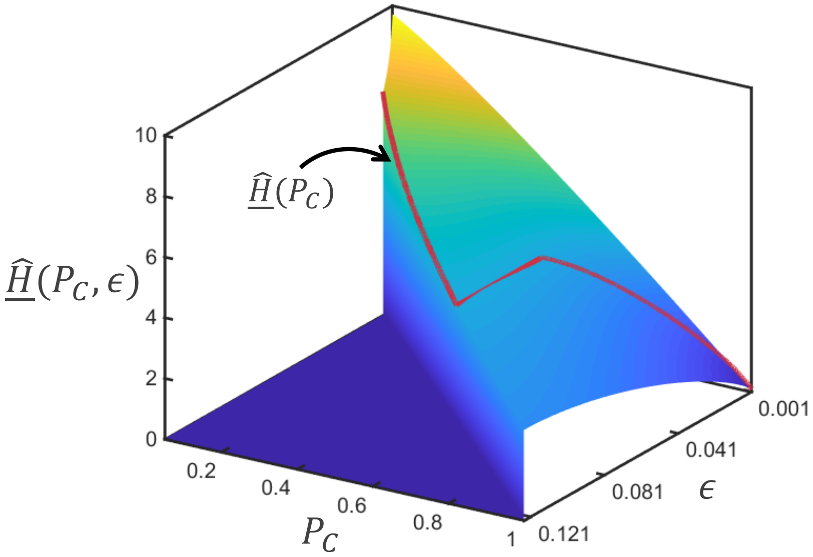

Supplementary Figure 23. Value of $\underline{\hat{H}}(P_C, \epsilon)$ and $\underline{\hat{H}}(P_C)$ with $C = 8$ in USAir network. The red solid curve denote the value of $\underline{\hat{H}}(P_C)$.

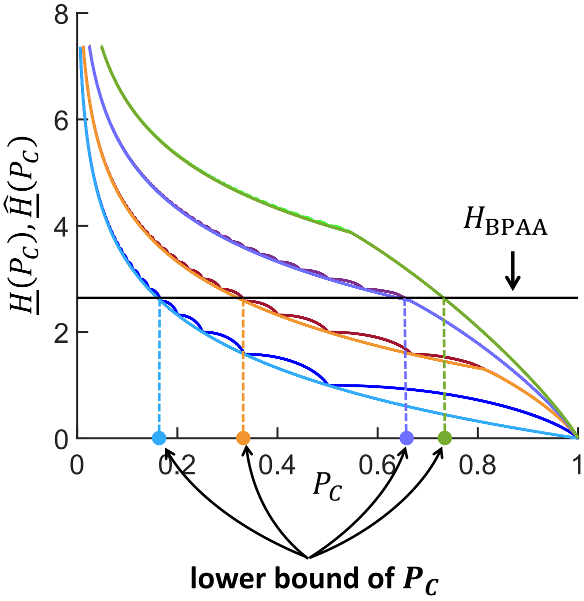

Supplementary Figure 24. Determination of the lower bound $\underline{P}_C$ in USAir network. The color dashed line represents the approximated value $\underline{\hat{H}}(P_C)$, while the solid line represents the real one $\underline{H}(P_C)$. The horizontal line denotes the value of $\underline{P}_C$.

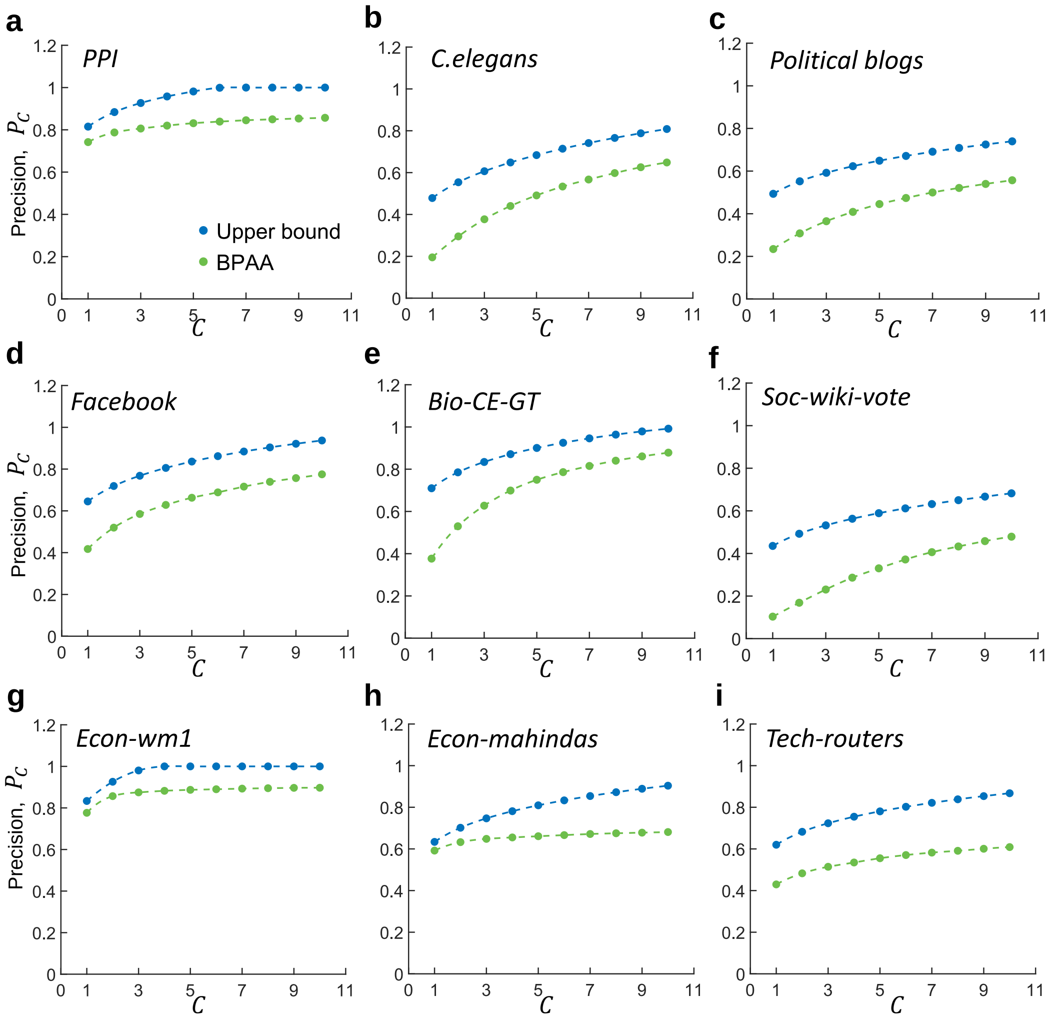

Supplementary Figure 25. Upper bounds of link prediction precision. The prediction prediction is defined by P_C that the missing link is in the top C intervals of rankings.

We can use the similar method to Supplementary Equation 105 to obtain an approximation $\underline{\hat{H}}(P_C)$ of $\underline{\hat{H}}(P_C, \epsilon)$. Supplementary Figure 23 give an illustration of $\underline{\hat{H}}(P_C)$ and $\underline{H}(P_C, \epsilon)$. As shown in Supplementary Figure 24, the difference between the value of $\underline{\hat{H}}(P_C)$ and $\underline{H}(P_C)$ is negligibly small especially when C is large. We also observe that $\underline{\hat{H}}(P_C)$ is decreasing with $\underline{\hat{H}}(P_C) = 0$ of $P_C = 1$. Therefore, there must exist a point of intersection for $\overline{H}(p_1)$ and the given $H_{\text{BPAA}} = (\log N - 1)H^*_{\text{BPAA}}$ in the range $[2C/N, 1]$. The P_C obtained from the intersection point is exactly the optimal solution $\underline{P}_C$ of the original problem.

$$\underline{P}_C = \min\{P_C : -\gamma(P_C, \epsilon)\log\gamma(P_C, \epsilon) - (1 - \gamma(P_C, \epsilon))\log\epsilon = (\log N - 1)H^*_{\text{BPAA}}\} \quad (107)$$

Results of upper bound on real-world networks

In Supplementary Figure 25, we provide results of upper bounds of link prediction precision for 9 empirical networks studied in this work, compared with the performance achieved by BPAA among 11 state-of-art algorithms described in Supplementary Note .

Distribution of p_j under the BPAA

In Supplementary Figure 26, we provide the distribution of p_j in the 12 networks studied with the BPAA, to directly investigate the validity of the assumption of $p_1 \geq p_2 \geq ... \geq p_{N/2}$.

Supplementary Note 11: Discussion on the data commercial value

In this section, we discuss in detail a general framework of the data commercial value for link predictability. Intuitively, the intrinsic value of a network dataset is minimal if it is deterministic and predictable, as the buyer of the data does not need to buy the data to know the structure inside. Our work focuses on the derived value of the networks: that is, the possible new links that can be predicted using the existing network dataset. In other words, we focus on the value of the potential or unobserved links using existing links, not existing links themselves. This could be the case for drug discovery, for instance, that one wants to predict new possible drugs using existing data; or recommendation systems for products that the particular consumer has never bought before.

In practice, when someone intends to pay for a network dataset, the price or value is related to how much additional commercial benefit can be retrieved from it. In general, there can be two situations: First, if there is no alternative information, and the buyer uses only the network data and develop prediction algorithms to benefit from the data, and say the amount of benefit is V; Second, if there are other information available that helps the buyer understand the network data even without having the data, the buyer can still have a baseline benefit from such external information, say Z amount. The later could be the case when a movie producer has some experience about the overall market without knowing who watches what types of movies. For instance, a movie producer may achieve available prediction results by simply recommending well-known popular movies with good box office to the audience. So, if the value Z is captured within V, the additional benefit from the dataset combined with prediction algorithm is $V - Z$ (see Supplementary Figure 27a).

Deterministic network like the lattice does not need much information to know its exact structure, and carries less value. For random networks like the ER network, it is not possible to know any better than random guess for

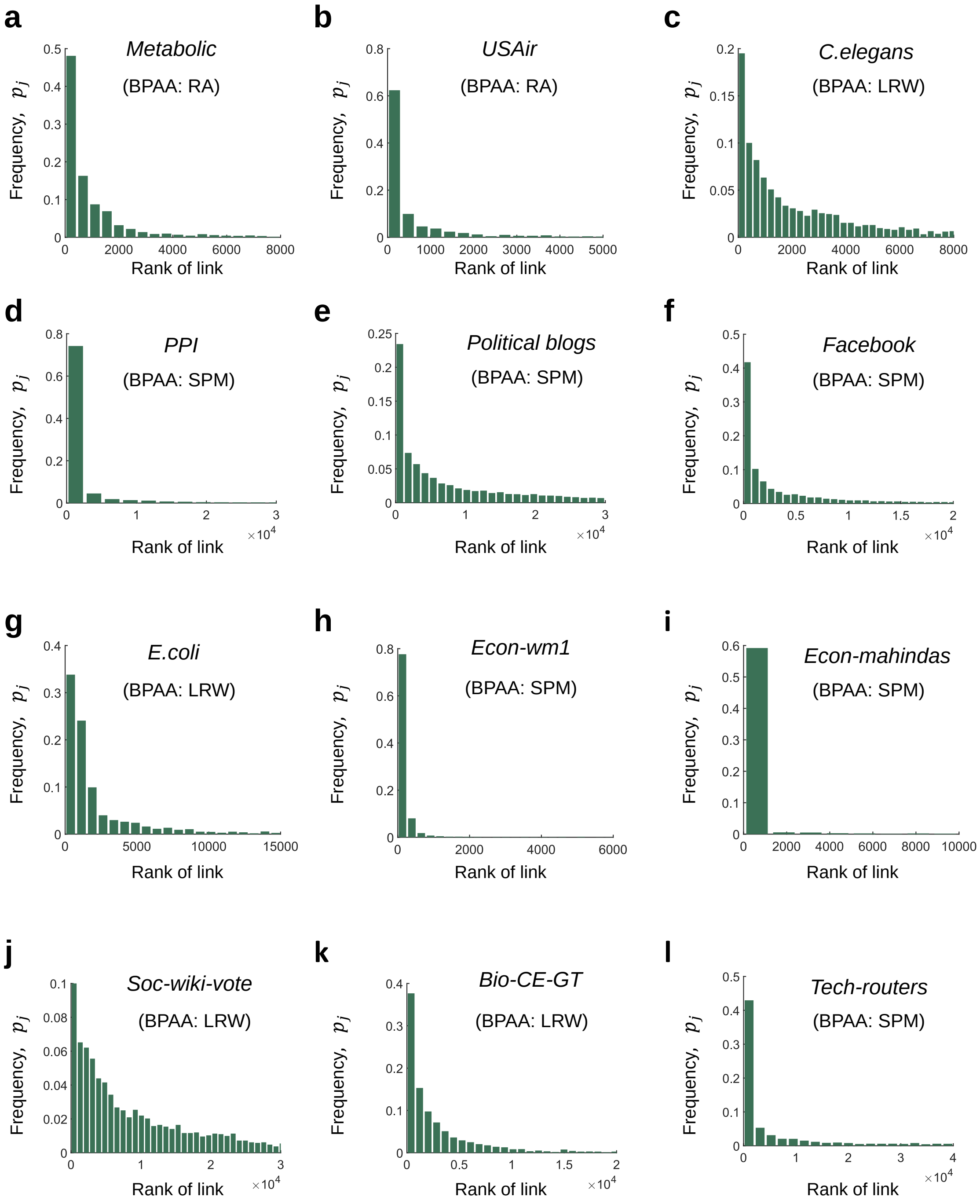

Supplementary Figure 26. Distribution of p_j in the 12 networks studied with the BPAA.

predicting links, and the value of the existing links is high, but the predictive value is low. Hence, we can assume that the value Z and V are related, say $Z = f(V)$. Next, we could quantify the additional value of link prediction, $W = V - f(V)$. There are different scenarios: (1) For $f(V) = 0$, corresponding to the case discussed in the main text. (2) For regular structure like the lattice, $f(V) = V$ as the network structure is fully captured by other known information. (3) For totally random network like the ER network, both $V = 0$ and $f(V) = 0$.

We next examine the 3 cases separately, as illustrated in Supplementary Figure 27b,c. (1) For the case of $f(V) = 0$ represented by the red line, there is no external information or such information does not overlap with the predictive benefit from the dataset. The additional value of the dataset is simple $W = V$. (2) For $f(V) = V$ in green, external information (i.e. knowing that it is lattice structure) leads to the value V equal to Z, when external information fully captures the network data. In such a case $W = 0$, and no additional value is provided by the dataset. (3) $f(V)$ increases together with V but smaller than V, shown in blue. Here the additional value from the dataset $W = V - f(V)$ is significant, and corresponds to many real problems when different information/dataset contributes to the total value.

The commercial value we refer to in Eqn. 12 in the main text is the potential additional value that can be realized by predicting unobserved information from the dataset. For example, in life science research, obtaining the protein-protein interactions needs extensive experimentations that cost both time and resources; if one can make predictions on such interactions to narrow the experimentation scope, it can reduce the cost of experiments, which is the predictive value of the existing dataset. A way to quantify such value is the cost per experiment multiplied by the reduction in the number of experiments to obtain accurate results. As we are not concerned or able to know the base value Z or $f(V)$ from information outside the network dataset, we focus only on the predictive value of the dataset in Eqn. 12 in the main text.

Supplementary References

[1] Watts, D. J. & Strogatz, S. H. Collective dynamics of ‘small-world’ networks. *Nature* **393**, 440 (1998).

[2] Jeong, H., Tombor, B., Albert, R., Oltvai, Z. N. & Barabási, A.-L. The large-scale organization of metabolic networks. *Nature* **407**, 651 (2000).

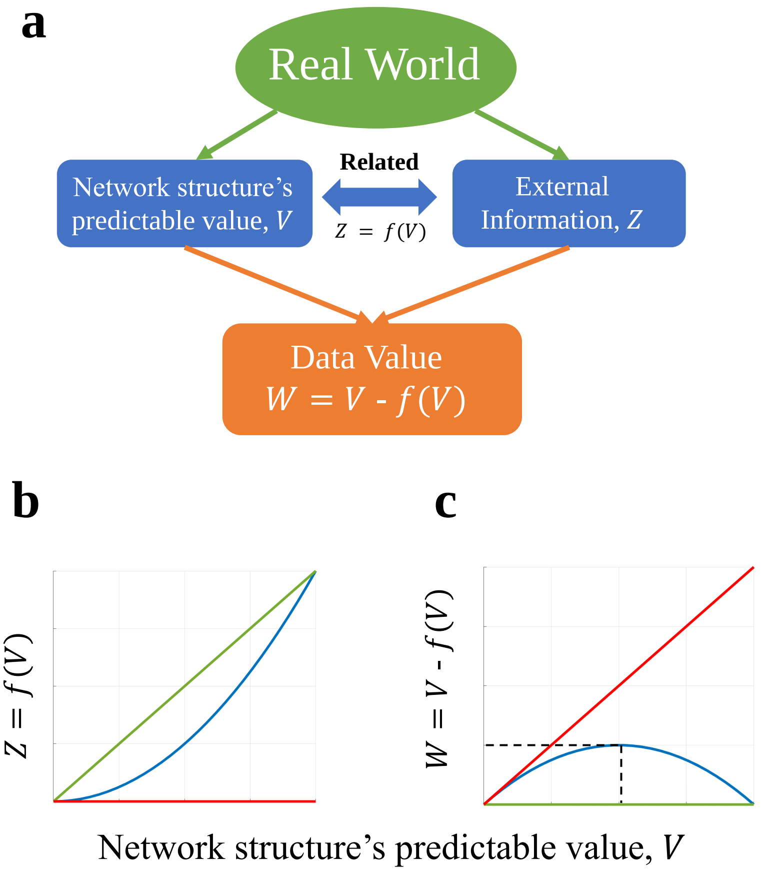

Supplementary Figure 27. Commercial value of the network dataset considering both its predictive value and its baseline value.

[3] Von Mering, C. *et al.* Comparative assessment of large-scale data sets of protein–protein interactions. *Nature* **417**, 399 (2002).

[4] Jin, Y., Turaev, D., Weinmaier, T., Rattei, T. & Makse, H. A. The evolutionary dynamics of protein-protein interaction networks inferred from the reconstruction of ancient networks. *PloS One* **8**, e58134 (2013).

[5] Rossi, R. A. & Ahmed, N. K. The network data repository with interactive graph analytics and visualization. In *Proceedings of the 29th AAAI Conference on Artificial Intelligence* (2015). URL `http://networkrepository.com`.

[6] Ackland, R. *et al.* Mapping the US political blogosphere: Are conservative bloggers more prominent. In *BlogTalk Downunder 2005 Conference, Sydney* (2005).

[7] Leskovec, J. & Krevl, A. SNAP datasets: Stanford large network dataset collection (2014). URL `http://snap.stanford.edu/data`.

[8] Batagelj, V. & Mrvar, A. Pajek datasets (2006). URL `http://vlado.fmf.uni-lj.si/pub/networks/data`.

[9] Spring, N., Mahajan, R., Wetherall, D. & Anderson, T. Measuring ISP topologies with rocketfuel. *IEEE/ACM Trans. Netw.* **12**, 2–16 (2004).

[10] Choi, Y. & Szpankowski, W. Compression of graphical structures: Fundamental limits, algorithms, and experiments. *IEEE Trans. Inf. Theory* **58**, 620–638 (2012).

[11] Skretting, K., Husøy, J. H. & Aase, S. O. Improved Huffman coding using recursive splitting. In *Proceedings of Norwegian Signal Processing*, 92–95 (1999).

[12] Cover, T. M. & Thomas, J. A. *Elements of information theory* (John Wiley & Sons, New York, 2012).

[13] Breiman, L. & Spector, P. Submodel selection and evaluation in regression. the x-random case. *Int. Stat. Rev.* 291–319 (1992).

[14] Kohavi, R. *et al.* A study of cross-validation and bootstrap for accuracy estimation and model selection. In *Processings of 15th International Joint Conferences on Artificial Intelligence*, 2, 1137–1145 (1995).

[15] Lü, L. & Zhou, T. Link prediction in complex networks: A survey. *Physica A* **390**, 1150–1170 (2011).

[16] Liben-Nowell, D. & Kleinberg, J. The link-prediction problem for social networks. *J. Assoc. Inf. Sci. Technol.* **58**, 1019–1031 (2007).

[17] Lü, L., Pan, L., Zhou, T., Zhang, Y.-C. & Stanley, H. E. Toward link predictability of complex networks. *Proc. Natl Acad. Sci. USA* **112**, 2325–2330 (2015).

[18] Liu, W. & Lü, L. Link prediction based on local random walk. *EPL* **89**, 58007 (2010).

[19] Klein, D. J. & Randić, M. Resistance distance. *J. Math. Chem.* **12**, 81–95 (1993).

[20] Fouss, F., Pirotte, A., Renders, J.-M. & Saerens, M. Random-walk computation of similarities between nodes of a graph with application to collaborative recommendation. *IEEE Trans. Knowl. Data Eng.* **19**, 355–369 (2007).

[21] Kovács, I. A. *et al.* Network-based prediction of protein interactions. *Nat. Commu.* **10**, 1240 (2019).

[22] Jaccard, P. Étude comparative de la distribution florale dans une portion des alpes et des jura. *Bull. Soc. Vaudoise Sci. Nat.* **37**, 547–579 (1901).

[23] Chowdhury, G. G. *Introduction to modern information retrieval* (Facet Publishing, London, 2010).

[24] Barabási, A.-L. & Albert, R. Emergence of scaling in random networks. *Science* **286**, 509–512 (1999).

[25] Xie, Y.-B., Zhou, T. & Wang, B.-H. Scale-free networks without growth. *Physica A* **387**, 1683–1688 (2008).

[26] Adamic, L. A. & Adar, E. Friends and neighbors on the web. *Soc. Netw.* **25**, 211–230 (2003).

[27] Zhou, T., Lü, L. & Zhang, Y.-C. Predicting missing links via local information. *Eur. Phys. J. B* **71**, 623–630 (2009).

[28] Kim, J. H., Sudakov, B. & Vu, V. H. On the asymmetry of random regular graphs and random graphs. *Random Struct. Algor.* **21**, 216–224 (2002).

[29] Karrer, B. & Newman, M. E. Stochastic blockmodels and community structure in networks. *Phys. Rev. E* **83**, 016107 (2011).

[30] Newman, M. E. & Peixoto, T. P. Generalized communities in networks. *Phys. Rev. Lett.* **115**, 088701 (2015).

[31] Waxman, B. M. Routing of multipoint connections. *IEEE J. Sel. Areas Comm.* **6**, 1617–1622 (1988).

[32] McEliece, R. *The theory of information and coding* (Cambridge University Press, Cambridge, 2002).

[33] Bertsekas, D. P. *Nonlinear programming* (Athena Scientific, MA, 1999).

[34] Boyd, S. & Vandenberghe, L. *Convex optimization* (Cambridge University Press, Cambridge, 2004).
